# Formulating sustainable planning for Goulan Yao Village based on the integration of cultural landscape gene theory and spatial analysis

**DOI:** 10.1038/s41598-025-15357-2

**Published:** 2025-08-14

**Authors:** Mengen Gu, Yajing Wang, Yang Wu, Yue Dai, Wei Fan

**Affiliations:** 1 School of Art, Hunan University of Information Technology, Changsha, 410151 China; 2 Hengdian College of Film & Television, Jinhua, 322118 China; 3 School of Fine Arts, Hunan Normal University, Changsha, 410012 China

**Keywords:** Traditional village landscape, Cultural landscape genes, Goulan Yao Village, Sustainable development, Environmental social sciences, Sustainability

## Abstract

Traditional village cultural landscapes, as vital carriers of human civilization, face severe threats amid rapid urbanization. This study centers on Goulan Yao Village in Jiangyong County, China, applying cultural landscape gene theory combined with GIS spatial analysis. A gene information chain map and a village-specific classification and coding system were established to analyze spatial distribution and organizational patterns. Results show that the village reflects a rich cultural connotation shaped by the natural environment, religious beliefs, and ethnic customs. Its landscape gene distribution exhibits a “three-core, multi-node” pattern with dense clustering and mismatches between spatial accessibility and concentration. Based on these insights, a sustainable planning framework of “connotation preservation–spatial optimization–cultural tourism innovation” is proposed. This framework aims to offer both theoretical support and practical guidance for cultural landscape conservation and rural revitalization of Yao ethnic villages. The study not only extends the application of cultural landscape gene theory to ethnic minority settlements but also provides a methodological reference for the sustainable development of similar traditional settlements worldwide.

## Introduction

Traditional settlements, as organic unities of natural and cultural heritage, embody profound historical memories and distinctive cultural values of human civilization^1^. From Indigenous settlements in the Americas, medieval towns in Europe, and tribal villages in Africa to traditional rural communities in Asia, these historic settlements collectively form a diverse mosaic of global cultural landscapes, reflecting the deep-rooted regional identities of different civilizations. However, with rapid urban expansion, accelerated industrialization, and the transformation of traditional ways of life, the ecological and cultural structures of traditional villages are under severe threat, with escalating landscape degradation and cultural discontinuity^2^. Data indicate that nearly 920,000 ancient villages in China have disappeared over the past two decades, and the rate of loss continues at approximately 1.6 villages per day^3^. In the context of global rural development and modernization, how to effectively safeguard and transmit these cultural carriers has become a critical global issue in cultural heritage preservation.

In recent years, the international community has shown increasing concern for the protection of traditional villages. UNESCO has inscribed numerous representative traditional settlements onto the World Heritage List, and governments worldwide have successively introduced policies and regulations to explore heritage preservation strategies tailored to their national contexts^4^. For instance, the European Union has incorporated rural landscape conservation into its 2020–2027 Common Agricultural Policy as a key agenda item^5^. Between 2012 and 2023, China designated six batches of nationally recognized traditional villages, totaling 8,155 entries, establishing the world’s largest agrarian cultural heritage system^6^. In the context of China’s rural heritage conservation, the terms “historical and cultural villages,” “traditional villages,” and “ethnic minority villages” denote distinct yet sometimes overlapping categories, each tied to specific policy frameworks. Historical and cultural villages refer to settlements officially designated by the State Council or provincial governments for their significant architectural and historical value, with emphasis on the preservation of built heritage^7^. In contrast, traditional villages are a broader category rooted in the concept of “ancient villages” from the 1980s, and were formally defined in a 2012 notice issued by four national ministries. They refer to early-established villages rich in traditional resources and recognized for their historical, cultural, scientific, and social value, thus warranting preservation^8^. Ethnic minority characteristic villages are typically located in specific geographic areas and are predominantly inhabited by a particular ethnic minority group. These villages effectively preserve and showcase the cultural traditions of their respective communities, serving as important windows for studying and experiencing ethnic minority cultures^9^. Goulan Yao Village, the subject of this study, embodies both a nationally recognized traditional village and a typical ethnic minority settlement. Therefore, it necessitates a hybrid analytical approach that accounts for both heritage preservation and ethnic spatial-cultural dynamics.

Although the classification systems and targeted protection policies for traditional and ethnic minority villages have made notable progress in recent years, many of these approaches still lack a systematic understanding of the deep cultural logic and spatial structures that shape these settlements. To address these challenges, the theory of cultural landscape genes offers a novel and integrative perspective. This theory analyzes the cultural characteristics of village landscapes to identify core elements marked by symbolism, continuity, and locality, extracting genetically stable “landscape genes.” These genes encompass not only tangible elements such as streets and buildings, but also intangible cultural components such as folk traditions and collective memory. Through in-depth analysis of the characteristics and internal logic of these genes, researchers can systematically uncover the underlying structures and evolutionary patterns of village cultural landscapes, providing a robust theoretical foundation for local landscape conservation and sustainable planning. While some scholars have conducted extensive studies in southwestern regions such as Hunan and Guizhou—particularly focusing on Miao^10^ and Dong villages^11^—existing literature tends to emphasize the extraction of local cultural symbols or typological classifications, with relatively little attention paid to the relational mechanisms between landscape genes, spatial structures, settlement morphology, and sustainable development planning. In particular, systematic studies of mountainous, defense-oriented settlements such as Goulan Yao Village remain limited.

Based on these questions, this study aims to establish a comprehensive landscape gene information chain for the Goulan Yao Village, encompassing both tangible and intangible cultural dimensions, and systematically identifying the components of their cultural landscapes. Utilizing GIS technology, the study delves into the spatial distribution and structural organization of landscape genes, uncovering the internal logic and spatial evolution mechanisms of cultural landscape elements in Yao settlements. It proposes planning strategies that balance traditional culture preservation, coordinated development of cultural tourism, and the activation of landscape genes, thus promoting the effective translation of theoretical results into actionable practices and building a closed-loop system linking theory and practice.

To achieve the above objectives, the study adopts a mixed-methods approach that integrates both qualitative and quantitative techniques. Qualitative methods, including in-depth interviews, non-participant observation, and ethnographic research, are used to identify the contents of landscape genes. These are complemented by quantitative approaches such as remote sensing interpretation, spatial accessibility analysis, and kernel density estimation, to comprehensively analyze spatial distribution patterns and clustering characteristics. The research establishes a bidirectional support mechanism between theoretical analysis and empirical validation, expanding the applicability of cultural landscape gene theory to mountainous, defense-oriented ethnic minority villages. This study also responds to global policy frameworks such as the United Nations Sustainable Development Goal 11, which emphasizes the creation of inclusive, safe, resilient, and sustainable human settlements^12^, and the UNESCO Convention Concerning the Protection of the World Cultural and Natural Heritage^13^. By focusing on the spatial analysis and planning of ethnic minority cultural landscapes, the research contributes to the integrated conservation of both tangible and intangible heritage, aligning local revitalization. It aims to provide paradigms and references for similar traditional villages in terms of cultural landscape preservation and sustainable development.

This paper is structured into six chapters: Introduction, Literature Review, Research Methodology, Research Results, Discussion, and Conclusion. The introductory section primarily presents the research background, objectives, and core questions, while Chapter 2 systematically reviews the relevant theoretical foundations and current research progress.

## Literature review

### Research progress on traditional village landscapes

Traditional villages refer to early-formed settlements that possess rich traditional resources, remain relatively well-preserved, and hold significant historical, cultural, scientific, artistic, social, and economic value^14^. As spatial carriers of “nature–culture” coupled systems, settlement landscapes embody the historical processes and cultural memories of specific ethnic groups adapting to natural environments. Through architectural forms, spatial structures, and ecological wisdom, they reveal a deep integration of local identity and ethnic characteristics, highlighting their co-evolution alongside human civilization. The conservation of traditional village landscapes is not only essential for preserving cultural diversity but also constitutes a key pathway to achieving rural sustainable development^15^.

The study of settlement landscapes has undergone multiple stages of development. As early as the mid-nineteenth century, German geographer Johann Georg Kohl systematically examined the spatial organization of settlements from a morphological perspective in his work *Transportation and Settlement of People and Their Dependence on Surface Terrain* ^16^. French geographer Paul Vidal de la Blache, in *Principles of Human Geography*, applied historical analysis to study the types, distributions, and interactions of rural settlements with agricultural systems^17^. In the latter half of the twentieth century, with the emergence of cultural heritage preservation ideologies, research focus shifted from historical descriptions to the recognition of the cultural value of landscapes, leading to greater thematic diversity. In the twenty-first century, research on settlement landscapes further expanded to include issues such as interactions between settlements and regional economies^18^, and landscape sustainability^19^. Some studies have also begun to explore multi-scalar spatial differentiation and morphological typologies of traditional settlements through GIS-based approaches. For example, analyses of the Nanxi River Basin in southern China have examined both the spatiotemporal evolution and cultural drivers of settlement heritage distribution, highlighting the shaping role of intangible heritage in settlement patterns^20^. Complementary morphological research on the same region has classified settlement types and extracted “mountain–water” spatial models using GIS and 3D visualization techniques, offering a framework for digital protection strategies^21^. These contributions enrich the empirical foundation for integrating spatial technology with cultural landscape research and offer replicable methods for comparable regions globally.

In recent years, research on traditional settlement landscapes has demonstrated a strong trend of interdisciplinary integration, with widespread incorporation of theories and methodologies from geography, sociology, anthropology, and history. Scholars have conducted in-depth studies on topics such as landscape typology^22^, land use^23^, spatial distribution^24^, spatiotemporal evolution^25^, and ecological transformation^26^. Substantial achievements have been made in methods of identification, evolution pathways, protection mechanisms, and transformation strategies, providing strong theoretical support for the inheritance and conservation of traditional settlements in the contemporary era.

In terms of practical conservation, the international community has explored diverse localized approaches to settlement landscape preservation. European countries jointly introduced the “European Landscape Convention”^27^, advocating for the systematic protection of both cultural and natural landscapes from a regional perspective, and promoting the extension of landscape preservation from elite culture to everyday life. In Japan, the Ministry of Agriculture, Forestry and Fisheries launched the “Redesigning Rural Landscapes Project”^28^, aiming to construct sustainable agricultural settlement systems through ecological restoration, landscape regeneration, and community participation. China has established a government-led, multi-stakeholder traditional village protection mechanism. Through policy tools such as the List of Chinese Traditional Villages, it has integrated heritage preservation into national spatial planning, thereby enhancing the systematic protection of landscape resources. Meanwhile, NGOs have actively promoted “eco-village” pilot projects^29^, emphasizing the integration of community self-governance and traditional ecological knowledge to facilitate the co-construction and sharing of landscapes and living systems. These diverse practices offer valuable experience for global traditional village conservation, particularly in institutional development, technical approaches, and public participation.

Despite significant advancements in settlement landscape research, notable limitations remain. Existing studies primarily focus on macro-level analyses of settlement morphology and functional structures or descriptions of physical forms, with limited attention to the deeper cultural logic—particularly intangible cultural heritage and other soft elements. Methodologically, most literature lacks systematic studies on the spatial correlations among cultural landscape gene elements and fails to establish analytical frameworks that reflect their distributional patterns and inherent logic, thereby constraining accurate identification of the underlying structure of cultural landscapes. Against this backdrop, the introduction of cultural landscape gene theory offers a novel theoretical lens and methodological tool for settlement landscape research. It attempts to extract key genetic information units from settlement landscapes to uncover their patterns of cultural transmission, evolution, and intrinsic structural characteristics. This provides both theoretical support and technical pathways for the living preservation and sustainable development of traditional settlements. This is of considerable significance for bridging current research gaps and enriching the theoretical framework for settlement landscape conservation.

### Development and application of the cultural landscape gene theory

The theory of cultural landscape genes is a comprehensive interdisciplinary approach that integrates knowledge from settlement geography, architecture, genetic biology, landscape morphology, and historical geography^30^. By incorporating symbolic visualization techniques and diagrammatic representation, this theory analyzes the spatial imagery of traditional settlement landscapes and constructs genetically meaningful landscape gene maps^31^. It has been widely applied in various fields, including traditional village conservation^32^, organic renewal^33^, architectural feature identification^34^, and tourism planning^35^. From its conceptual introduction to the gradual refinement of its theoretical system, the cultural landscape gene theory has entered a phase of rapid development.

The theoretical foundation of cultural landscape genes originates from the concept of “cultural genes” proposed by Richard Dawkins in *The Selfish Gene* (1976)^36^, which viewed culture as evolving through mechanisms similar to biological genes. In 1984, Nigel Taylor introduced the idea of “genes” into settlement landscape morphology studies^37^, laying groundwork for a morphological reading of cultural landscapes. Building on this foundation, Chinese scholar Liu Peilin formally introduced the concept of “cultural landscape genes”^30^ in 2003, integrating insights from historical geography, landscape ecology, and geoinformation mapping. He proposed that key cultural elements in traditional settlements exhibit unique, replicable, and mutable characteristics, similar to biological genes.

During its developmental stage (2003–2015), Liu and his team primarily focused on identifying and classifying landscape genes, establishing a theoretical system based on four core principles—internal uniqueness, external uniqueness, partial uniqueness, and overall dominance. Since 2015, research has gradually expanded to include more institutions and scholars, covering topics such as landscape perception^38^, restoration^39^, and intangible cultural heritage preservation^40^, with continual refinement of identification processes, influencing factors, and atlas construction principles (see Table 1). For example, Zou Weihan et al. developed a landscape gene identification system in their study of traditional settlements along the Shudao Road in southern Shaanxi, focusing on elements, patterns, structures, and symbolic meanings^41^. Hu Zui et al. constructed an index system based on 2D and 3D structures, visual, and perceptual elements^42^, proposing an object-oriented identification pathway to support the scientific classification and recognition of landscape genes. These studies have significantly advanced the theoretical maturity and methodological toolkit of cultural landscape gene research, facilitating its wider application in heritage assessment, spatial planning, and cultural landscape conservation.

**Table 1:** Overview of landscape gene research themes and methods.

Researchers	Research topic	Methodology	Case study area	Findings
Zhai et al.^43^	Identification of traditional settlement landscape features	Landscape gene mapping	Shaanxi Province	Identifying and extracting landscape cultural genes can reflect the basic characteristics of local regional culture
Li et al.^44^	Protection and restoration of traditional settlements	Participant observation, in-depth interviews	Huangdu Village, Hunan Province	Traditional villages achieve cultural inheritance and spatial revitalization through landscape symbol reconstruction
Jin et al.^47^	Evolution and variation of landscape gene morphology	Field interviews, grounded theory	Hekou Village, Gansu Province	Capital, elites, and conceptual changes jointly induce the variation of ancient town landscape genes
Li et al.^45^	Factors influencing tourist perception and place identity	Questionnaire survey	Qiqiao Village, Nanjing City	Differences in host-guest perception reveal that residents prioritize culture while tourists focus on layout in landscape gene evaluation
Sun et al.^46^	Protection of agricultural cultural heritage	GIS spatial analysis, participatory rural appraisal	Chaozhou City, Guangdong Province	Increased perceived benefits significantly enhance residents’ enthusiasm for protecting agricultural cultural landscape heritage
Liu et al.^47^	Landscape spatial planning and transformation	GIS spatial analysis, space syntax	Linpu Village, Fujian Province	Proposed an “extraction-construction-analysis-preservation” model to optimize the spatial pattern of Linpu Village’s landscape
Hu et al.^48^	Health evaluation mechanism of settlement landscape genes	AHP hierarchical analysis	Hengyang Area, Hunan Province	Natural environment, tourism development, architectural history, and management mechanisms are the four major factors affecting the health of traditional settlements

In tracing the theoretical development of cultural landscape gene research, it is worth noting that its underlying logic shares affinities with certain international morphological traditions. For example, while Liu Peilin’s framework emphasizes the uniqueness, mutability, and symbolic replicability of spatial elements within traditional settlements^49^, this line of inquiry also resonates with Aldo Rossi’s notion of urban typology, particularly his emphasis on the persistence of spatial form and collective memory^50^. Both perspectives reflect a concern for the continuity of place-based identity through time. However, the gene-based approach advances this further by proposing a quasi-biological mechanism of transmission and heredity, which is particularly suited for analyzing rural, vernacular, and minority landscapes. This is because such landscapes often retain more complete cultural transmission systems and exhibit relatively stable patterns of spatial evolution. As a result, the genetic characteristics, variation rules, and mechanisms of cultural elements are more identifiable and traceable. This intersection of indigenous theoretical innovation and global discourse provides a foundation for deeper cross-cultural engagement in the study of traditional settlement landscapes.

In recent years, the theory of cultural landscape genes has entered an application-oriented phase, increasingly integrated into traditional village conservation, spatial planning, tourism development, and intangible cultural heritage protection. A growing number of studies have explored landscape gene identification, variation mechanisms, and spatial evolution, contributing to a more refined understanding of traditional settlement systems. Technological innovations—including GIS-based analysis, remote sensing, digital modeling, and digital twin technologies—have enhanced the precision and efficiency of gene recognition and visualization. Emerging tools such as BIM and AI-assisted classification further expand the practical potential of the theory in digital conservation and intelligent planning.

However, despite these advances, current applications remain uneven. Most existing studies continue to emphasize gene extraction and symbolic representation, while systematic spatial analyses—particularly at the multidimensional, quantitative level—are still limited. Moreover, although theoretical frameworks are increasingly robust, the actual integration of gene-based insights into operational planning and sustainable development remains limited. Regional imbalances remain evident: although recent years have seen increased attention to the cultural landscape heritage of traditional settlements in southern China—such as Dong^51^ and Miao^10^ villages—research specifically focusing on Yao ethnic villages remains limited in both breadth and depth, particularly regarding the integration of landscape gene theory, spatial analysis, and sustainable development planning strategies. Some studies have begun to address relevant themes: for example, Cao et al.^52^ utilized the ArcGIS platform to construct a graphic database of landscape gene symbols for traditional settlements in southern China, covering ethnic types such as Tujia, Miao, Dong, Yao, and Hakka. While this offers useful insights for the development and application of regional-scale landscape gene atlases, the study focuses primarily on surface-level documentation and lacks in-depth spatial analysis and planning tailored to specific ethnic contexts. Similarly, Dou et al.^53^ explored memory place identification and landscape restoration in Goulan Yao Village using an integrated method combining landscape gene theory with big data analysis of Weibo images and texts. This offers valuable methodological insights into linking spatial memory and landscape restoration, yet it does not fully incorporate a gene-based spatial analysis framework or planning-oriented modeling approaches. Therefore, comprehensive investigation into the spatial logic and gene clustering of Yao settlements—especially from a planning and sustainable development perspective—remains underexplored. As one of the third-batch “Distinctive Minority Villages of China,” the Goulan Yao Village in Jiangyong County retains an intact traditional architectural form and spatial organizational logic, serving as a representative model of mountainous defensive settlement landscapes with strong ethnic cultural characteristics. Against the backdrop of national policies promoting rural revitalization and the protection of ethnic minority cultural heritage, as well as global agendas such as the UNESCO Convention Concerning the Protection of the World Cultural and Natural Heritage, there is an urgent need for systematic research on the village to inform its conservation and sustainable revitalization.

In recent years, an integrative spatial analysis perspective has gradually emerged, combining cultural landscape research with technologies such as Geographic Information Systems (GIS), space syntax, and spatial statistical modeling. This methodological convergence enables a more comprehensive understanding of how landscape genes function across spatial hierarchies and how they interact with settlement morphology and environmental variables. For example, Deng et al. developed a GIS-based gene management system for traditional settlements^54^, which provides a structured platform for the visualization, categorization, and dynamic monitoring of cultural landscape genes. Zhou et al. employed space syntax to quantify the spatial integration of Langtou Village and proposed accessibility-oriented design strategies^55^. Huang et al. used a combination of interaction detection, kernel density analysis, and other spatial techniques to study the rural settlement forms, centroid migration, and clustering patterns in the Nanxi River Basin^56^, offering insights into how cultural heritage preservation can be balanced with sustainable economic development.

Notably, such integrated approaches are also being adopted in countries beyond China, including Italy and South Korea. For instance, Statuto applied landscape metrics and spatial analysis tools—along with modern cartographic techniques and recent remote sensing imagery—to detect the natural evolution of a representative rural landscape in the Basilicata Region of southern Italy over the past 188 years, quantitatively assessing its transformation^57^. Benedetti^58^ combined place syntax analysis with the equivalent income line model to explore spatial equity and structure in peripheral rural areas of Italy, offering new perspectives for spatial optimization. In South Korea, Sun Hak Bae used geotagged images from Google Panoramio and geographic information technology to analyze the spatiotemporal density of tens of thousands of landscape photos in rural areas^59^, demonstrating the effectiveness of this method for investigating the spatial distribution of rural landscape heritage.

These research advances not only reflect an increasing convergence of interdisciplinary methods, but also demonstrate the unique strengths of GIS-based spatial analysis in visualizing landscape gene distributions, uncovering spatial clustering and accessibility patterns, and informing targeted conservation and planning strategies for traditional rural settlements.

This study is grounded in cultural landscape gene theory and takes the Goulan Yao Village as a typical case. It combines field surveys, semi-structured interviews, feature deconstruction, and historical tracing methods. Based on an analysis of the village’s production and lifestyle evolution, it systematically identifies its cultural landscape genes and constructs a spatial gene information chain. By introducing spatial analytical methods, the study further reveals the distribution characteristics and layout patterns of the Yao village’s landscape genes. It then explores the practical applications of these findings in spatial optimization and protection decision-making within settlement planning. The aim is to provide theoretical support for the systemic protection and dynamic cultural transmission of Yao traditional villages. It also serves as a methodological reference and source of practical inspiration for tourism planning and sustainable development in similar settlements.

## Research methodology

### Study area

**Fig. 1:**
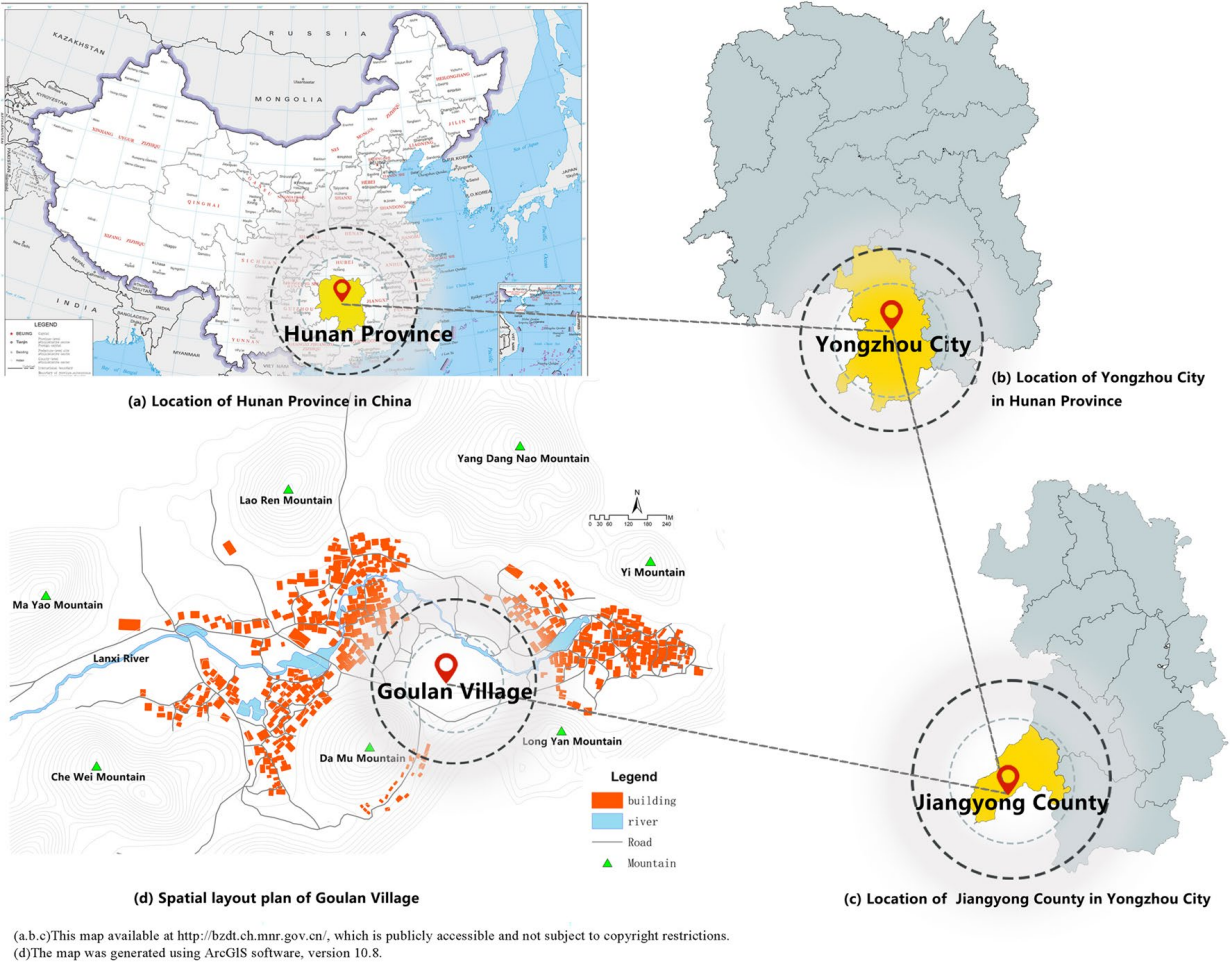
Location and Overall Landscape of Goulan Yao Village. Map generated by the author using ArcGIS 10.8 (Esri Inc., https://www.esri.com/en-us/arcgis/products/arcgis-desktop/overview).

Goulan Yao Village is located in Jiangyong County, at the southwestern border of Yongzhou City, Hunan Province, China. As the principal settlement of the Goulan Yao people—one of the “Four Major Yao Ethnic Groups”—the village is characterized by a castle-like mountain settlement pattern, naturally enclosed by mountains on all sides, which forms a robust defensive spatial structure. The village covers an area of approximately 6 square kilometers and administratively comprises three sub-villages: Shangcun, Xiacun, and Daxing, with a permanent population of around 3,000 residents. Established during the Ming Dynasty, the village possesses a profound historical and cultural legacy. The Lanxi River flows through the site, shaping a typical feng shui layout of “backed by mountains, encircled by water, and facing a natural screen”^60^ (see Fig. 1), reflecting the site selection wisdom of the Yao ancestors in adapting to mountainous environments. The traditional economic structure was mainly agrarian, supplemented by commerce and handicrafts. Over generations, the village has evolved into a multi-layered settlement system. More than 300 traditional residential structures from the Ming and Qing Dynasties remain, including defensive architecture such as village walls, night-watch houses, clan gatehouses, and alley portals, as well as public spaces for religious, ancestral, and educational functions—forming a composite and stratified Yao landscape system.

Beyond major Han Chinese festivals such as the Spring Festival and Mid-Autumn Festival, the Yao people celebrate unique ethnic festivals including the Panwang Festival, Bullfighting Festival, and Mud Washing Festival, which serve as key carriers of ethnic identity and cultural continuity. In 2019, Goulan Yao Village was selected as part of the third batch of “Ethnic Minority Characteristic Villages of China” and the eighth batch of China’s National Key Cultural Heritage Protection Units. What distinguishes Goulan Yao Village from other settlements in southern China lies in its rare integration of mountain fortress-type defensive spatial morphology, clan-based social organization, and ritual-based landscape logic, all preserved in a relatively complete and continuous form. Unlike many minority villages that have experienced spatial fragmentation, homogenization, or modern restructuring, Goulan retains a multi-core cluster settlement layout adapted to mountainous terrain, with spatial hierarchies clearly reflecting ritual order, kinship systems, and defensive needs. Its complex landscape system—comprising fort walls, watchtowers, ancestral halls, and sacred spaces for the Panwang Festival and the Mud Washing Festival—exemplifies a living model of cultural resilience in marginal mountain regions. This unique convergence of defense, belief, and dwelling in a single spatial ecology makes Goulan an exemplary case for testing and extending the cultural landscape gene theory in underexplored ethnic regions. However, with accelerating socio-economic transformation and tourism-driven development, the village faces growing challenges including the deterioration of historic buildings, functional decline of spatial systems, and the dilution of landscape character^61^. Addressing the tension between cultural heritage preservation and modern development has thus become an urgent and complex issue.

### Research methods

**Fig. 2:**
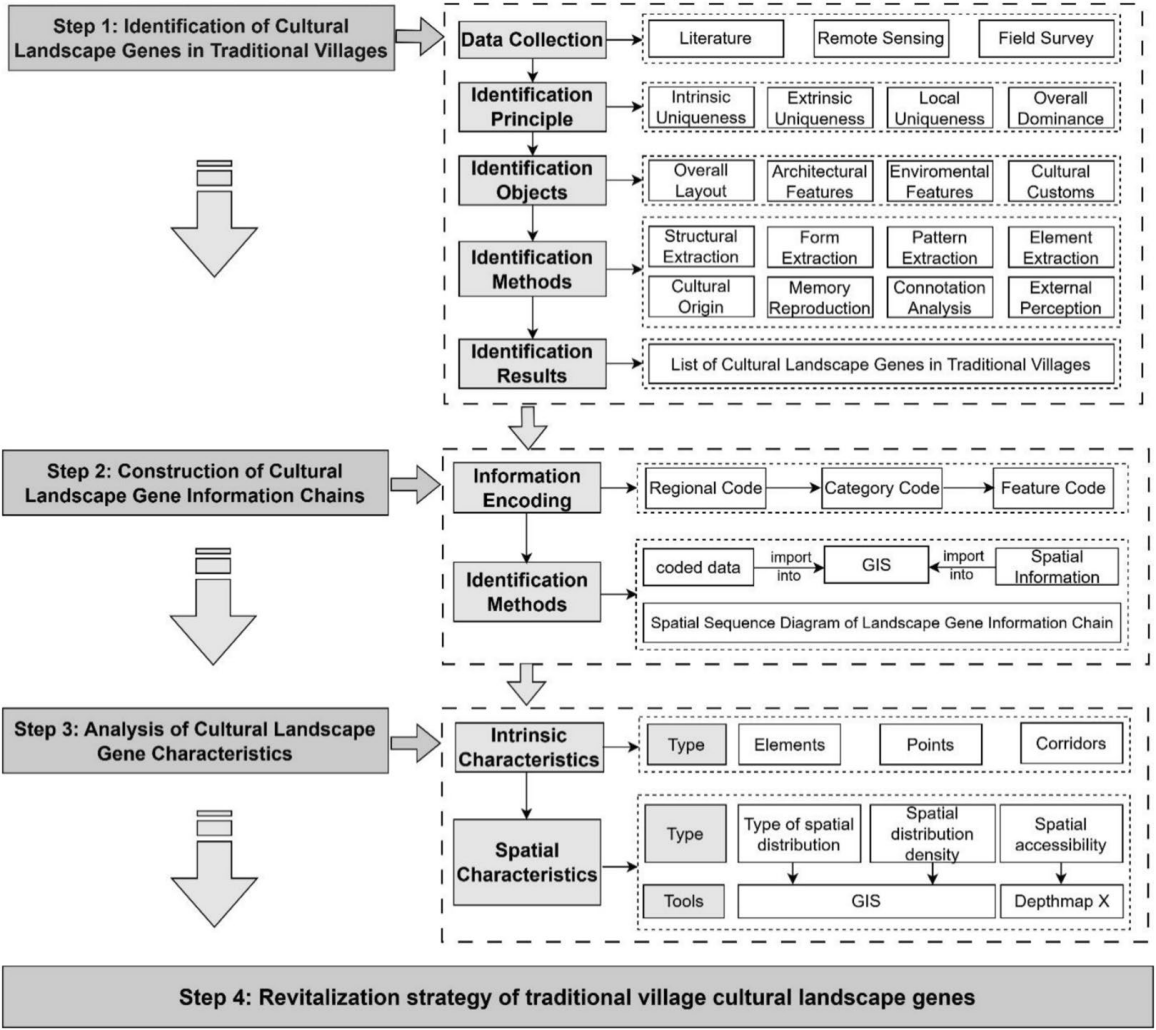
Research flow chart.

This study is structured around four core phases—extraction, construction, analysis, and transformation—to systematically build a cultural landscape gene information chain for Goulan Yao Village and propose operational strategies for sustainable planning and design. First, comprehensive data collection was conducted through field investigation, remote sensing imagery interpretation, and historical literature review. Based on established landscape gene identification principles and methodologies, the study identified and refined the core landscape genes applicable to Goulan Yao Village. A classification and coding system was then constructed to encompass both tangible and intangible elements, forming a visualized “landscape gene information chain” that captures the symbolic and structural logic embedded within the cultural landscape. Next, the study applies quantitative spatial analysis techniques—including GIS spatial tools, landscape element density indices, and space syntax—to explore the spatial distribution, correlation characteristics, and clustering patterns of various gene elements. By integrating the outcomes of gene identification and spatial analysis, the study proposes a set of sustainable planning strategies encompassing landscape conservation, spatial optimization, and cultural tourism integration (see Fig. 2). These strategies provide both theoretical support and practical guidance for revitalizing traditional settlements through evidence-based planning and culturally grounded development.

#### Identification and encoding of cultural landscape genes in Goulan Yao Village

The identification of cultural landscape genes in traditional settlements refers to the micro-level extraction of key representative elements that define the essence of local cultural landscapes. The methodological foundation of this study builds upon well-established academic frameworks and employs a mature theoretical lineage. Specifically, the study adopts the Feature Deconstruction Extraction Method proposed by Hu^42^, which classifies landscape genes into two main categories: material genes (including environmental and architectural features) and intangible genes (covering customs, clan systems, dialects, and religious beliefs). In accordance with the Guidelines for Strengthening the Protection of Chinese Traditional Villages^62^, the identification system was refined into nine main categories and twenty-four indicators (see Table 2). For material genes, the study follows structural, formal, and elemental extraction paths, including 11 evaluative indicators that are categorized into three groups: overall layout, architectural characteristics, and environmental components. For intangible landscape genes, the focus is placed on cultural context tracing, memory reconstruction, semantic interpretation, and sensory perception, covering six key domains: religious and ritual practices, traditional craftsmanship, performing arts, festivals and folk activities, social practices and customs, and language and oral traditions. This categorization allows for a more comprehensive understanding of the Yao community’s intangible heritage system, reflecting both its spiritual worldview and everyday cultural expressions. The identification process relies primarily on on-site fieldwork and visual observation, supplemented by interviews, site documentation, and historical textual analysis. This comprehensive approach enables a systematic recognition of both natural and cultural features within the settlement landscape^63^.

**Table 2:** Cultural landscape gene identification system for Goulan Yao village.

Gene category	Identification factor	Identification sub-indicators
Material cultural genes	Overall layout	Village form
		Street layout
		Fluvial form
		Landscape pattern
	Architectural features	Plane layout
		Public building
		Architectural style
		Architectural decoration
		Architectural texture
	Environmental elements	Built environment
		Natural environment
	Religious and ritual practices	Panwang Festival
		Bird Worship Festival
		Shamanic culture
Non-material cultural genes	Traditional craftsmanship	Yao Embroidery
	Performing arts	Long Drum Dance
		Lusheng Dance
		Seated Song Hall Singing
	Festivals and folk activities	Mud Washing Festival
		Ox King Festival
	Social practices and customs	Clan-based communal living
		Matrilocal marriage
	Language and oral traditions	Yao language
		Yao folk mountain songs

#### Method for constructing the landscape gene information chain

**Fig. 3:**
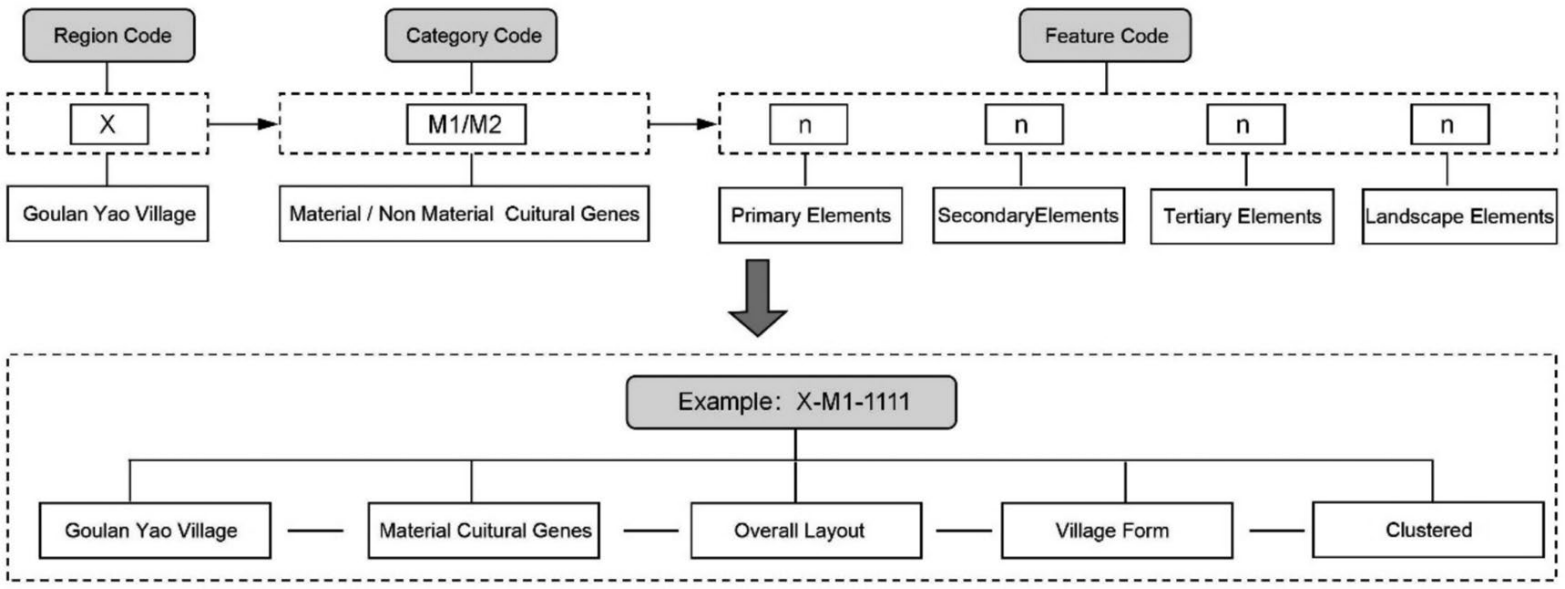
Coding structure of cultural landscape genes.

To accurately classify and interpret the kinship relationships among various local cultural landscape genes, and to systematically record and manage the relevant data, it is essential to sequence and encode the identified landscape genes, thereby constructing a landscape gene information chain map. The coding method in this study is based on established techniques cited in several key studies^64,65^, which have been successfully applied by other researchers in similar contexts. Typological principles were adopted in combination with N-level coding theory to encode the traditional settlement landscape gene information of Goulan Yao Village^66^. The coding structure for cultural landscape genes is divided into regional codes, category codes, and feature codes. The regional code for Goulan Yao Village is designated as “X,” and the category codes are labeled “M,” referring to “Meme” based on Richard Dawkins’ concept^36^, representing cultural landscape genes. Further, material culture genes are denoted as M1, and intangible culture genes as M2. Feature codes are assigned following the Classification and Coding of Fundamental Geographic Information Features^65^, wherein the hierarchical structure of gene subordination is divided into four levels: primary element, secondary element, tertiary element, and landscape gene element. Arabic numerals are used for coding (see Fig. 3). The encoding of Goulan Yao Village’s cultural landscape genes was undertaken by researchers with extensive experience in relevant fields. To ensure the accuracy and consistency of the coding results, two team members independently completed the initial coding phase. Their results were then compared and analyzed, and any discrepancies were resolved through discussion and negotiation. Subsequently, senior researchers reviewed and validated the final codes to ensure compliance with established standards and research specifications.

#### Method for spatial feature analysis of cultural landscape genes

To deeply explore the spatial characteristics of cultural landscape genes in Goulan Yao Village, this study integrates multiple analytical tools, including spatial distribution type analysis, density evaluation, and accessibility quantification. Through these technical approaches, it becomes possible to reveal the organizational structure and distribution patterns of landscape genes, assess the clustering intensity of cultural values, and optimize spatial accessibility—thus providing a scientific basis for conservation strategies and sustainable development planning. All spatial maps in this study were generated by the author using ArcGIS 10.8 (Esri Inc.) and DepthmapX 0.8.0 (developed by UCL Bartlett Space Syntax). ArcGIS was used for spatial data processing and visualization, while DepthmapX was employed for space syntax analysis.

##### Method for analyzing the spatial distribution types of genes

To evaluate the spatial organization of cultural landscape genes, it is first necessary to determine their distribution type in order to formulate targeted conservation and management measures. Accordingly, this study adopts the Average Nearest Neighbor (ANN) method for analysis. From a macro perspective, the cultural landscape gene units of Goulan Yao Village appear as point elements, whose spatial distribution patterns can be categorized into three types: random, clustered, and uniform. The ANN method is commonly used to determine the spatial distribution mode of point elements and is also suitable for analyzing the spatial distribution characteristics of cultural landscape genes^67^.

The formula for calculating the ANN ratio is as follows:

1 $$ANN=\frac{\begin{array}{c}\overline{{D}_{O}}\end{array}}{{D}_{E}}$$

2 $${D}_{E}=\frac{1}{\sqrt[2]{n/A}}$$

where $\overline{{D}_{O}}$ represents the actual average distance between each cultural landscape gene unit and its nearest neighbor; ${D}_{E}$ denotes the expected average distance assuming a random distribution; $N$ is the total number of landscape gene units; and $A$ is the area of the village (encompassing all cultural landscape gene units). When $ANN$=1, the distribution is random; when $ANN$>1, the genes tend to be dispersed; and when $ANN$<1, the genes exhibit a clustered distribution pattern.

##### Method for analyzing the spatial density of genes

To scientifically identify the core areas of landscape cultural heritage and develop differentiated conservation strategies, this study analyzes the spatial density distribution of landscape genes. High-density regions often indicate clusters of cultural heritage, which are critical for formulating zoning-based conservation strategies and optimizing resource allocation. Therefore, kernel density analysis is employed to assess spatial density, a technique commonly used for analyzing the spatial density of point features and applicable to cultural landscape gene studies^68^. In the density calculation process, weights are assigned based on the number of gene elements each spatial unit contains. For units with multiple cultural landscape genes, the weight is adjusted proportionally, calculated as the ratio of the number of genes in a unit to the total number of genes across all units. This weighted approach ensures that units with higher concentrations of genes exert a greater influence on the overall density estimation and helps highlight zones of heightened cultural and historical significance^69^.

The formula for weighted kernel density ($W\_KD$) is as follows:

3 $$W\_KD(x)=\frac{1}{{\sum }_{i=1}^{n}{W}_{i}}\sum_{i=1}^{n}{W}_{i}K\left(\frac{x-{X}_{i}}{h}\right)$$

where $x$ denotes the estimated density at a specific location; ${W}_{i}$ is the weight of the $\text{i}$-th unit, equal to the proportion of cultural landscape genes it contains relative to the total; $K$ is the kernel function; ${X}_{i}$ represents the coordinate of the $i$-th landscape gene unit; and $h$ is the search radius of the kernel function.

Using ArcGIS 10.8 (developed by Esri Inc., Redlands, California, USA), a spatial distribution model of Goulan Yao Village’s cultural landscape genes was constructed. Genetic elements were quantified, and kernel density analysis was performed to identify spatial distribution patterns. The results were visualized in map form, with gene density levels categorized based on unit distribution characteristics. In the generated maps, color depth reflects kernel density values, with darker tones indicating higher density concentrations.

##### Method for analyzing gene spatial accessibility

Spatial accessibility evaluation aims to quantify the potential of cultural landscape genes to attract human flows, thereby assessing their dissemination and display capacity within the traditional village context^70^. Generally, areas with high accessibility are more likely to form visitor aggregation zones. Within the spatial structure of a traditional village, various cultural landscape genes are interconnected through existing transport pathways, forming a spatial network with internal logic. The accessibility of different landscape gene units within the network is influenced by their positional relationships and the complexity of connection paths. To assess movement costs within the spatial network, the concept of topological depth is introduced: the higher the topological depth value, the longer the path required to reach the unit from other points in the network, and the lower its accessibility.

Accordingly, this study adopts Global Integration as a key metric to evaluate the accessibility of cultural landscape genes. This metric calculates the inverse of the sum of topological depths between a given gene unit and all others, indicating its level of spatial integration. Generally, higher integration implies better spatial connectivity and enhanced potential for cultural gene expression and functional activation^71^. In practice, the spatial axial line network of Goulan Yao Village was analyzed using Depthmap X 0.8.0, developed by the Space Syntax Laboratory at The Bartlett School of Architecture, University College London. This analysis identified critical nodes of cultural landscape genes within the spatial structure based on global integration values. The corresponding formula is as follows:

4 $${I}_{i}=\frac{\left(n-2\right){D}_{n}}{2\left(n{Di}^{-1}\right)}$$

where *MD*_*i*_ is the mean topological depth of cultural landscape gene *i*, calculated as:

5 $${MD}_{i }=\frac{{TD}_{i}}{n-1}=\frac{{\sum }_{j-1}^{n}{d}_{ij}}{n-1}$$

6 $$Dn =\frac{2\left\{n\left[\mathit{log}\left(\frac{n+2}{3}\right)-1\right]+1\right\}}{\left(n-1\right)\left(n-2\right)}$$

Here, *n* represents the total number of cultural landscape genes in the historical village; *D*_*N*_ denotes the average distance between landscape genes; *TD*_*i*_ is the total depth of gene *i*, the sum of the steps required to reach all other genes; and d_*ij*_ is the distance from gene *i* to gene *j*.

### Data sources

The data sources for this research mainly include the following three aspects:

#### Field investigation

From March to October 2024, the research team conducted multiple field surveys in Goulan Yao Village. Through interviews, non-participant observation, GPS mapping, and image acquisition, comprehensive data were collected regarding the village’s historical development, cultural and natural landscapes, land use transformation, and spatial structure. A total of 23 semi-structured interviews were conducted using a purposive sampling strategy to ensure comprehensive representation of key stakeholder groups. The sampling criteria included: (1) long-term residents with diverse backgrounds including general villagers and shop owners; (2) village committee members with administrative knowledge including current and former leadership; (3) scenic area staff representing tourism industry perspectives; (4) out-of-town visitors representing external perspectives; and (5) intangible cultural heritage inheritors to capture traditional cultural knowledge. Interview participants included villagers (n = 13, comprising 8 general villagers, 3 shop owners, and 2 intangible cultural heritage inheritors), village committee members (n = 2, including current village secretary and retired former village secretary), scenic area staff (n = 3, including ticketing staff, cultural tour guide, and hotel receptionist), and tourists (n = 5, all out-of-town visitors), focusing on topics such as the evolution of the village, types of cultural landscapes, and the transformation of traditional architecture and road systems (see Table 3). Theoretical saturation was achieved after 20 interviews, with the final three interviews yielding no new themes or insights, confirming data sufficiency.

**Table 3:** Information of interview subjects and interview focus.

Code	Interview group	Number of participants	Specific identity description	Main focus of interview
A1–A13	Villagers	13	A1–A8: General villagers, A9–A11: Shop owners, A12–A13: Intangible cultural heritage inheritors	Village development history and perception of cultural landscape
B1–B2	Village committee members	2	B1: Current village secretary, B2: Retired former village secretary	Village management, cultural heritage protection policies and measures
C1–C3	Scenic area staff	3	C1: Ticketing staff, C2: Cultural tour guide, C3: Hotel receptionist	Operation of scenic area, tourism services and cultural dissemination practices
D1–D5	Tourists	5	D1–D5: Out-of-town visitors	Cultural experience and perception of landscape attraction

Ethical approval for this study was granted by the Biomedical Research Ethics Committee of Hunan Normal University. Prior to participation, informed consent was obtained from all interviewees, including villagers, village committee members, tourism staff, and visitors. Participants were fully informed about the purpose of the study, the voluntary nature of their participation, and their right to withdraw at any time. All data collected were anonymized to ensure confidentiality and privacy. All methods were performed in accordance with relevant guidelines and regulations, and the research was conducted in accordance with the Declaration of Helsinki. Each interview ranged from 20 to 90 minutes. These first-hand materials provide a robust empirical basis for the in-depth identification and interpretation of the characteristics and evolutionary logic of the cultural landscape genes in Goulan Yao Village.

#### Documentary data

To ensure the historical depth and reliability of the study, in addition to retrieving relevant academic literature from databases such as CNKI, Wanfang, and Google Scholar, this research systematically reviewed Yongming County Annals and Jiangyong County Annals^72^, focusing on sections concerning natural environments and human geography. Local inscriptions, poems, and other historical documents were also examined for cross-verification. Genealogical records from local families were analyzed to gain a detailed understanding of the village’s ethnic composition and early developmental trajectory. Furthermore, planning documents from official government websites and media platforms were incorporated, including the Goulan Yao Village Tourism Development Master Plan, Goulan Yao Village Historic Cultural Village Conservation Plan, and Beautiful Countryside Construction Plan of Goulan Yao Village^73^. These materials were systematically categorized and interpreted using documentary analysis methods, providing a solid textual foundation for understanding the spatial distribution of cultural landscape genes.

#### Remote sensing imagery

Basic spatial data were sourced primarily from the National Geomatics Center of China and the Geospatial Data Cloud^74^, supplemented by platforms such as Google Earth and Global Mapper. Remote sensing imagery and contour terrain data of the Goulan Yao Village area were acquired. Using ArcGIS 10.8 software, features such as buildings, roads, and water systems were vectorized. The collected imagery was processed through mosaicking and geometric correction. Combined with field survey results, these data facilitated a comprehensive spatial analysis of the village’s landscape characteristics and land use changes.

## Results

### Identification and coding of cultural landscape genes

#### Identification and extraction of cultural landscape genes in Goulan Yao Village

Research on the identification of cultural landscape genes reveals that Goulan Yao Village possesses a multi-layered and diverse set of cultural landscape characteristics (see Table 4). In terms of spatial patterns, the village exhibits a layout surrounded by hills and paddy fields, a linear river system, a spine-like street structure, and a clustered village organization. Architecturally, the village features traditional buildings primarily from the Ming, Qing, and Republican periods, including religious structures, defensive facilities, and ancestral halls. Decorative elements such as wood carvings, stone carvings, and grey plaster are widely used, reflecting the artistic and symbolic richness of traditional Yao architecture.

**Table 4:** Results of cultural landscape gene identification in Goulan Yao Village.

Gene category	Identification factor	Identification sub-indicators	Results
Material cultural genes	Overall layout	Village form	Clustered
		Street layout	Spinal form
		Fluvial form	Throughflow type
		Landscape pattern	Hillside-Field Ring
	Architectural features	Plane layout	Single-courtyard
			Courtyard-well
		Public building	Religious buildings
			Defensive structures
			Ancestral halls
			Leisure structures
			Educational buildings
		Architectural style	Roof types
			Gable wall types
			Standing windows
		Architectural decoration	Decoration locations
			Decoration techniques
			Decoration themes
		Architectural texture	Colors
			Materials
	Environmental elements	Built environment	Forest of steles
			Spiral well
			Stone village wall
		Natural environment	Ancient tree
			Natural pond
Non-material cultural genes	Religious and ritual practices	Panwang Festival	
		Bird Worship Festival	
		Shamanic culture	
	Traditional craftsmanship	Yao Embroidery	
	Performing arts	Long Drum Dance	
		Lusheng Dance	
		Seated Song Hall Singing	
	Festivals and folk activities	Mud Washing Festival	
		Ox King Festival	
	Social practices and customs	Clan-based communal living	
		Matrilocal marriage	
	Language and oral traditions	Yao language	
		Yao folk mountain songs	

Regarding environmental features, the cultural heritage landscape comprises human-made elements such as stone steles, defensive stone walls, and ancient wells, while also relying on natural elements like old trees and waterways to form an integrated eco-cultural framework. At the intangible level, the cultural genes of Goulan Yao Village encompass a wide array of traditional practices and expressions. These include religious and ritual practices such as the Panwang Festival and Bird Worship Festival; traditional craftsmanship exemplified by Yao embroidery; performing arts like Long Drum Dance, Lusheng Dance, and Seated Song Hall Singing; as well as distinctive folk festivals such as the Mud Washing Festival and Ox King Festival. The village also retains unique social practices and customs, including clan-based communal living and matrilocal marriage systems, alongside the preservation of the Yao language and oral traditions such as mountain ballads. These intangible heritage elements have developed over centuries as adaptive responses to the village’s mountainous geography, clan-based social organization, and animistic cosmology. Seasonal cycles, agricultural rituals, and intergenerational transmission within kinship units have collectively shaped a vibrant and resilient cultural landscape system.

#### Construction and visualization of the cultural landscape gene information chain

**Fig. 4:**
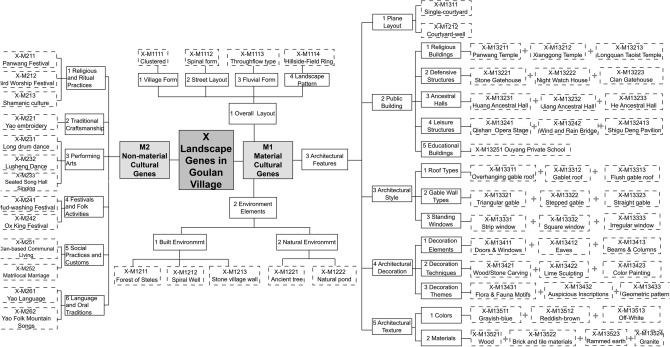
Landscape gene information chain map of Goulan village.

The research team adopted an independent coding approach for data analysis. Results showed a high degree of consistency between the two coders. A small number of classification differences were noted, primarily due to subjective interpretations of specific landscape features. To resolve these discrepancies, a thematic workshop was held during which the team reached consensus through systematic discussion and deliberation. As a result, the coding standards were unified and the accuracy of the results was ensured. Figure 4 presents several examples of cultural landscape gene coding, which demonstrate the stability and broad applicability of the adopted coding framework. To enhance intuitive understanding, Table 5 presents selected representative cultural heritage elements of Goulan Yao Village, visually supporting the interpretation of the coding results. The finalized coding results comprehensively and systematically depict the classified characteristics of Goulan Yao Village’s cultural landscape genes. These results were reviewed and validated by a panel of senior experts, guaranteeing the scientific rigor and reliability of the classification system.

**Table 5:** Representative cultural heritage elements of Goulan Yao Village.

No	Element Name	Heritage Description	Image
1	Hillside-Field Ring	Defensive layout protected by encircling mountains	
2	Courtyard-well	“Four-water-returns-hall” wealth-gathering *feng-shui*	
3	Panwang Temple	Ancestral shrine central to Yao identity	
4	Clan Gatehouse	Clan boundary marker and defensive gate	
5	Huang Ancestral Hall	Core space symbolising clan lineage	
6	Shigu Deng Pavilion	Landmark pavilion for rest, view, and shelter	
7	Stone village wall	Stone walls merged with terrain as first defense	
8	Yao Embroidery	Totemic embroidery expressing female identity	
9	Long Drum Dance	Vigorous drum dance symbolising Yao valor	
10	Mud Washing Festival	Post-ploughing agrarian carnival and rites	

**Fig. 5:**
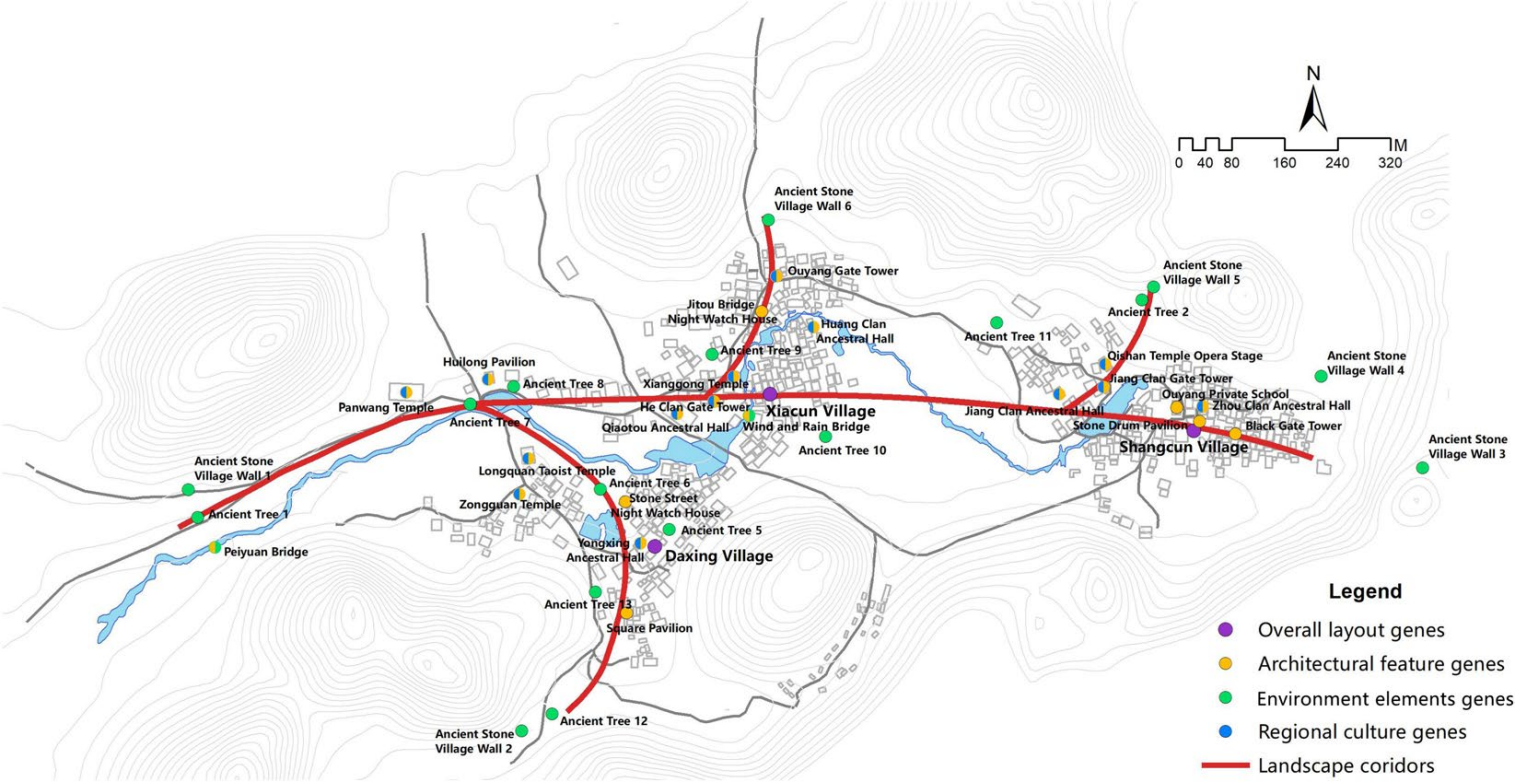
Spatial sequence diagram of cultural landscape gene information chain in Goulan village. Map generated by the author using ArcGIS 10.8 (Esri Inc., https://www.esri.com/en-us/arcgis/products/arcgis-desktop/overview).

Based on Geographic Information System (GIS) technology, the study conducted spatial localization analysis of cultural landscape gene elements, extracting key parameters such as ID labels, code data, and geographic coordinates. The spatial distribution of the cultural landscape genes was then visualized in vector format (see Fig. 5). Analytical results show that the cultural landscape gene information chain of Goulan Yao Village consists of four landscape corridors, representing the spatial manifestation of the landscape information chain^75^. One primary corridor runs east–west, while three secondary corridors extend north–south. The street system forms a typical spine-like structure, with a central main road serving as the organizing axis around which major architectural complexes are arranged. Branch roads and courtyards line both sides of this main route, creating a rhythmic village layout that reflects the overall organizational logic of settlement genes. Genes related to architectural characteristics and regional cultural traits are mainly concentrated in the eastern and central-western areas of the village, whereas environmental reference genes are primarily distributed in the northern and southwestern parts. This spatial configuration is primarily shaped by the village’s mountainous terrain, which channels movement along valley axes and restricts expansion to linear corridors. Meanwhile, the concentration of architectural and cultural genes in central areas reflects the Yao people’s social structure, which prioritizes communal gathering and ritual centrality, resulting in a layered yet compact spatial gene distribution pattern. This spatial pattern analysis provides crucial insights into the composition and evolution of the cultural landscape in the region.

### Analysis of the content characteristics of the landscape gene information chain in Goulan Yao Village

**Fig. 6:**
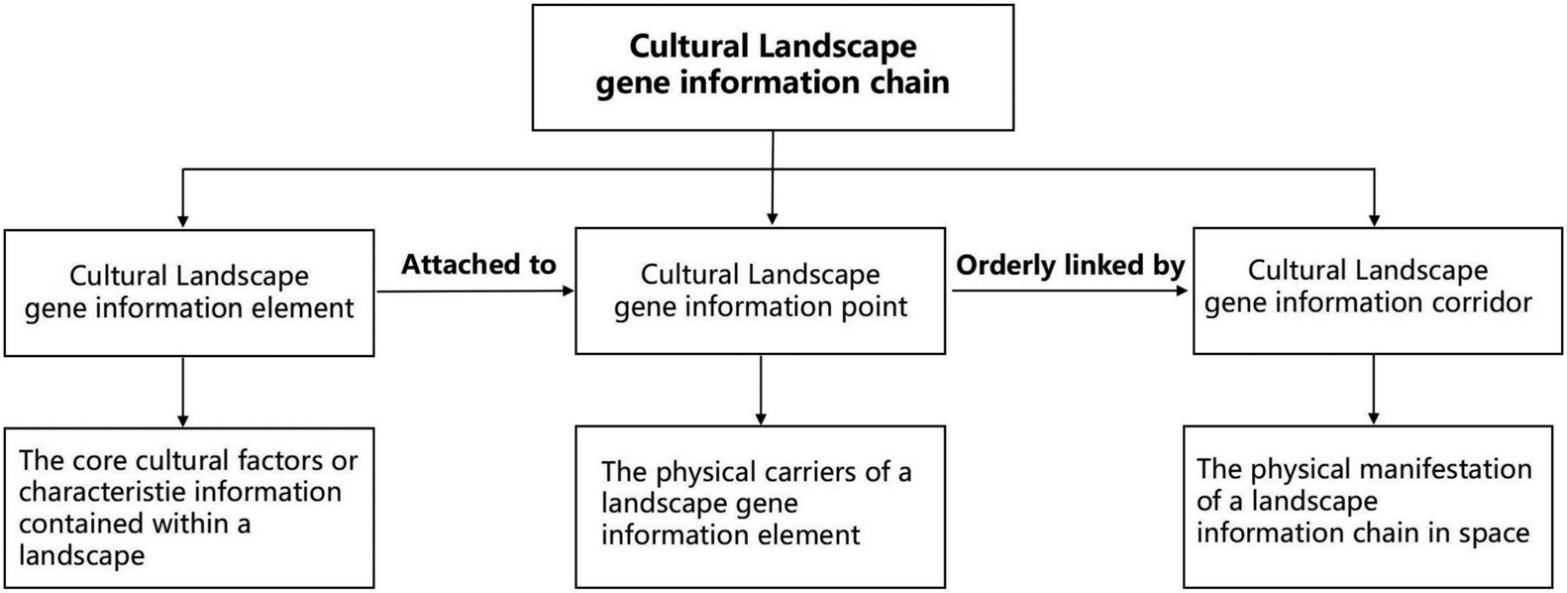
Internal composition of cultural landscape gene information chain.

The landscape gene information chain comprises three key components: information elements, information nodes, and information corridors^75^. Among them, information elements refer to the intrinsic cultural factors embedded within the local landscape, such as the natural environment, religious beliefs, and customary practices. Information nodes are the material manifestations of these elements, expressed in tangible forms such as residential buildings, principal public structures, and referential spatial landmarks within streets and alleys—they serve as physical carriers of the embedded cultural elements. Together, landscape information elements and nodes constitute the basic components and units of the landscape information chain. In contrast, information corridors represent the spatial expression of the landscape gene information chain. Based on the identification and extraction of Goulan Yao Village’s cultural landscape genes (Table 4), a gene chain (Fig. 4) and vector map (Fig. 5) were constructed, from which the core characteristics of the information elements, nodes, and corridors were derived, as shown in (Fig. 6).

#### Characteristics of cultural landscape gene information elements

From an environmental perspective, Goulan Yao Village is situated amidst mountains and water, adhering closely to feng shui principles and exhibiting a high degree of spatial integration with the natural landscape. Nine stone gate towers form an enclosed structure, reflecting the residents’ psychological need for security. The Lanxi River flows through the village, and numerous bridges and pavilions create a unique streetscape described as “walking three miles to see four pavilions, water flowing a hundred steps to cross ten bridges.”Yao religious beliefs are rooted in the animistic worldview of the ethnic group, resulting in a diversified belief system centered on ancestor worship, nature worship, and polytheism. Temples are widespread, and ritual activities are abundant, forming the spiritual core of the community. In terms of folk culture, the village preserves a wealth of intangible cultural heritage and traditional festivals, such as the Panwang Festival, Mud Washing Festival, and Ox King Festival. During these events, villagers perform Long Drum Dance, sacred bull rituals, and martial arts to pray for blessings, demonstrating a rich ethnic identity. These cultural elements are deeply embedded in the local context and constitute the unique cultural landscape genes of the area. However, the village is currently facing significant challenges in cultural heritage preservation. The weakening of clan consciousness has led to a decline in traditional rituals such as ancestral worship^76^. Additionally, important intangible heritage practices like Long Drum Dance and Yao Embroidery are at risk due to the aging of practitioners and low youth participation. Certain valuable cultural elements and crafts have yet to receive adequate attention, placing the village at risk of losing its cultural diversity^77^.

#### Characteristics of cultural landscape gene information nodes

Goulan Yao Village contains a wide range of landscape information nodes, primarily in the form of architectural structures. The village buildings can be categorized into residential dwellings, public buildings, and defensive structures.

Residential dwellings are primarily distributed along both sides of the Lanxi River, mainly consisting of brick-and-wood courtyard houses facing east or oriented westward. Their spatial typology includes both single courtyards and atrium courtyards, with the latter being dominant. The layout of these dwellings lacks a clear central axis and follows natural topography, resulting in winding, staggered, and enclosed alley spaces. Architectural decoration is centered on wood carving, supplemented by stone carving and grey plaster sculpture, with decorative motifs that are dynamic, expressive, and freely composed. Relief carvings, roof animals, and carved brackets often appear on doors and lintels. Roofs are typically gabled, and the buildings are strongly enclosed—first floors are usually windowless, relying on atriums for lighting. The gable designs vary, including horizontal, shouldered, and gable-headed forms, which vividly express ethnic identity and contribute to a dynamic and unique street facade.

Public buildings primarily include ancient stages, study halls, temples, and ancestral halls from the Ming and Qing dynasties. The Panwang Temple Stage served as a central space for cultural activities. The Shigu Pavilion is a classic example of a three-story, sixteen-column wooden gatehouse with upturned eaves, embodying the Yao aspiration for prosperity. Ancestral halls, mainly from the Ming dynasty, serve dual roles in religious rituals and administrative affairs^78^. However, many public buildings have suffered from neglect, structural damage, and decay, raising concerns about their preservation.

Defensive structures are among the most distinctive features of Goulan Yao Village^61^. The internal clan-based residential system gave rise to compound courtyards protected by four layers of fortifications: gatehouses, passage gates, night-watch towers, and stone walls. These nodes are typically located at turning points or central areas of the alley network. The village also contains over 70 bridges, including wooden beam bridges, stone slab bridges, and bluestone arch bridges, with the Peiyuan Bridge, built in the early Qing Dynasty, being the most representative. The Wind and Rain Bridges serve not only as shelter from the elements but also as communal gathering and resting spaces. Together, these elements form the rich architectural expression of Goulan Yao Village’s cultural landscape.

#### Characteristics of cultural landscape gene information corridors

The landscape information corridor system in Goulan Yao Village consists of a primary corridor and several secondary corridors, forming an integrated spatial network. The primary corridor runs in a Y-shaped formation along the east–west axis and serves as the village’s core spatial spine. It links major nodes such as the stone walls, Panwang Temple, Huilong Pavilion, Xianggong Temple, Xinglong Temple, Bengshan Temple, and Shigu Pavilion. This corridor not only connects religious, cultural, and defensive spaces but also functions as the main route between the village’s two natural subunits. The secondary corridors are primarily distributed within the sub-villages, linking building clusters with the main axis. They typically connect through defensive nodes such as night watch houses and passage gates. The entire corridor network resembles a tree structure: the Y-shaped primary corridor functions as the trunk, and the branching secondary corridors represent the limbs, with various buildings—residences, temples, and ritual spaces—attached as leaves. This results in an organic spatial structure with qualities of growth and continuity, establishing a cohesive, multi-level, and dynamic system for cultural information transmission.

However, due to age-related deterioration and a lack of systematic maintenance, parts of the corridor system are broken or damaged, significantly compromising both functionality and visual continuity. The unregulated insertion of modern infrastructure further disrupts the traditional landscape. Elements such as concrete pavements, electric cables, and communication facilities differ drastically from the historical context in appearance, weakening the corridor system’s unity and undermining the continuity and expressive power of the original spatial fabric. Additionally, peripheral corridors leading to nodes such as ancient wells and stone steles lack adequate signage and systematic integration. As a result, the visibility and communicative power of certain cultural landscape elements are low, and their potential value remains underutilized—placing them at risk of marginalization and neglect. Therefore, it is urgent to implement comprehensive corridor planning and aesthetic control to enhance the integrity and sustainability of cultural expression across the village.

### Spatial characteristics analysis of cultural landscape genes in Goulan Yao Village

#### Spatial distribution type analysis of cultural landscape genes

**Fig. 7:**
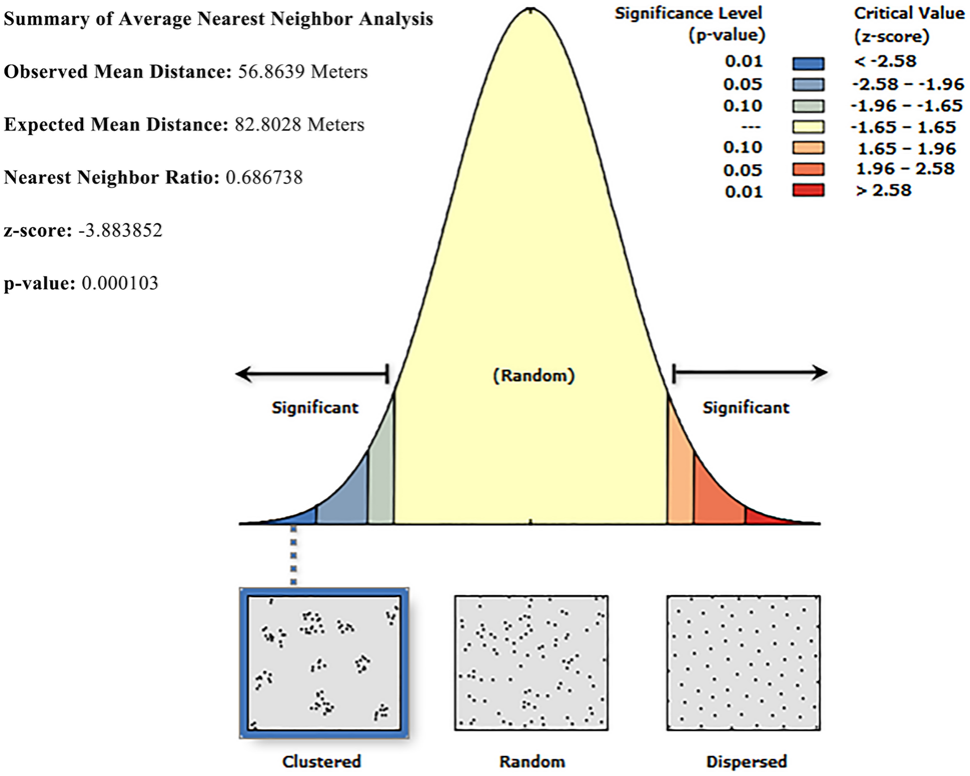
Average nearest neighbor analysis of cultural landscape genes in Goulan Yao Village.

To explore the spatial distribution characteristics of cultural landscape genes in Goulan Yao Village, this study conducted quantitative analysis using the Average Nearest Neighbor (ANN) method based on the ArcGIS platform. The results (Fig. 7) show that the ANN ratio for cultural landscape genes in the study area is 0.686 (ANN < 1), indicating a significantly clustered spatial distribution pattern (p < 0.01). This clustering phenomenon is largely influenced by natural geographic conditions. Goulan Yao Village is located in the karst landform region of southern Hunan, situated in a semi-enclosed basin surrounded by mountains. Constrained by terrain, most buildings are laid out along hillsides, rivers, or traditional post roads, and the three natural sub-villages form clustered residential zones along the valley. This terrain-adaptive spatial organization facilitates the spatial aggregation of cultural landscape genes. On one hand, it allows visitors to experience the core elements of Yao culture within a compact area. On the other hand, the high-density use of spatial resources causes crowding among cultural landscape elements, leaving insufficient transition and buffer zones. As a result, the boundaries between different landscape units become blurred, making it difficult to highlight their unique cultural identities and values. This not only diminishes the visitor experience but also hinders rational allocation and sustainable use of cultural landscape resources.

#### Spatial density analysis of cultural landscape genes

**Fig. 8:**
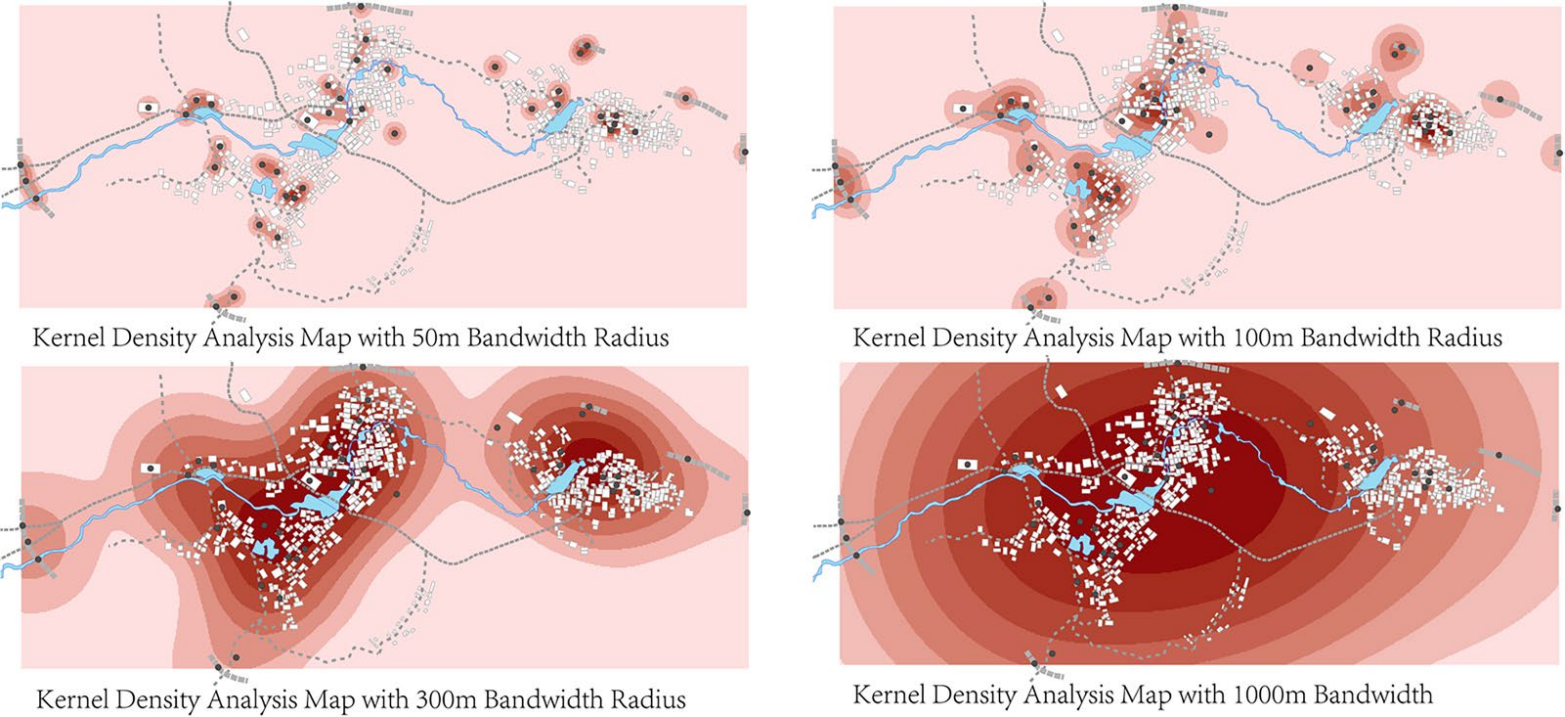
Kernel density with varying bandwidths (50, 100, 300, 1000 m).

Given the relatively dense distribution of cultural landscape element points in Goulan Yao Village and the small spatial scale of the study area, selecting an appropriate search radius is crucial for preserving spatial detail in the kernel density analysis. To evaluate sensitivity, this study tested four different search radii—50 m, 100 m, 300 m, and 1000 m—and compared their kernel density output effects. The results indicate that larger radii tend to produce overly smoothed surfaces, leading to boundary distortion and masking of local clustering patterns, whereas smaller radii (e.g., 50 m) generate overly fragmented results with scattered hotspots that are difficult to interpret at the village scale. Among the four options, the 100-m radius best balances resolution and readability, effectively revealing the core clustering structures of cultural landscape nodes while preserving micro-scale spatial characteristics. Accordingly, 100 m was selected as the optimal search radius, and the output raster resolution was set to 3 m. A visual comparison of the kernel density outcomes across different radii is presented in Fig. 8, further supporting the parameter choice.

**Fig. 9:**
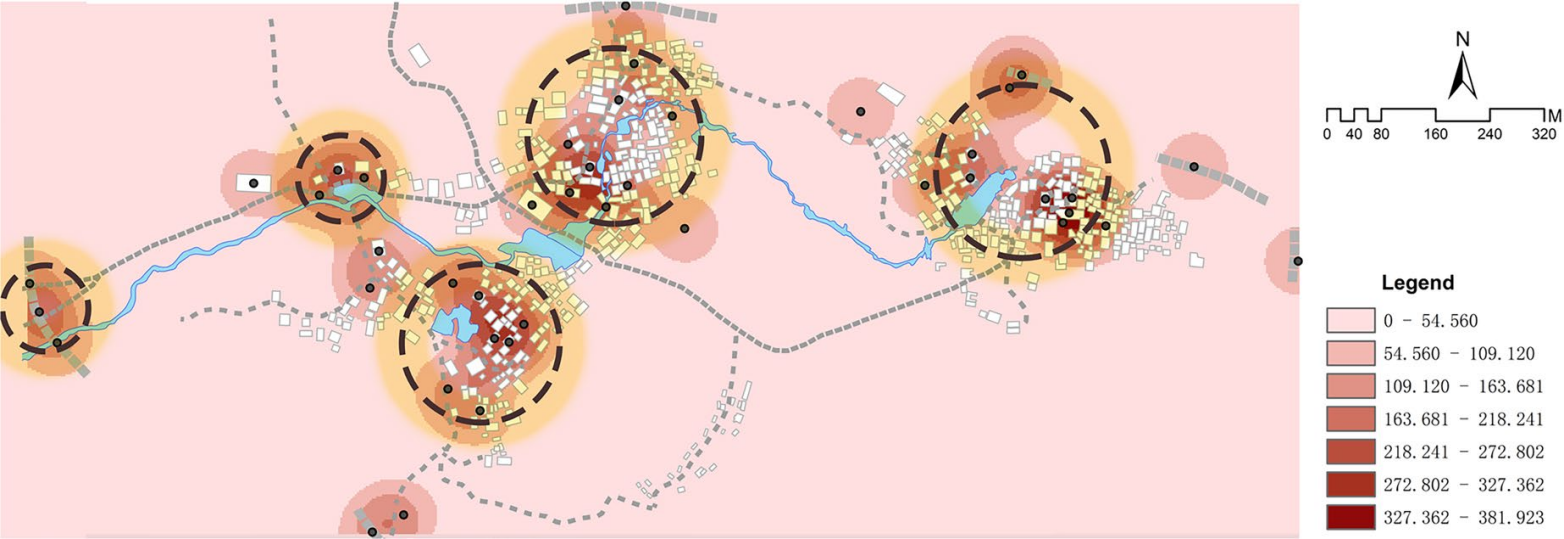
Kernel density distribution characteristics of landscape information points in Goulan Yao Village. Map generated by the author using ArcGIS 10.8 (Esri Inc., https://www.esri.com/en-us/arcgis/products/arcgis-desktop/overview).

The kernel density calculation results demonstrate that the density of landscape information points in Goulan Yao Village exhibits a multi-core aggregation pattern, specifically a “three-core multi-point nesting” model (Fig. 9). The kernel density map in Fig. 9 uses a seven-level color scale to represent the spatial clustering intensity of landscape information points. The color bands range from light pink (Level 1, 0–54.560) to dark red (Level 7, 327.362–381.923). Each level corresponds to a specific numerical interval, providing an intuitive visualization of the spatial distribution strength of settlement landscape genes. The three highest-density regions are located in the centers of the three natural village units and in the public activity spaces along the river. The highest density values reached Level 7 (327.362–381.923), represented by dark red coloration indicating significant clustering. Many key landscape nodes—such as the ancestral hall at the bridgehead, Xianggong Temple, Ouyang Academy, Wind and Rain Bridge, and Black Gate Tower—are located within these zones. Two secondary clusters were identified in the western part of the village: one is the “gateway core area” composed of Peiyuan Bridge and the ancient stone wall, and the other is the “ancestral temple cluster area” formed by Panwang Temple and Huilong Pavilion. Their density values ranged from Level 2 to Level 5 (54.560–272.802), representing secondary cultural landscape cores. Outside these concentrated areas, the distribution of cultural landscape genes in peripheral zones was more scattered. Density values were mostly within Levels 1 to 3 (0–163.681), with light pink coloration, and the genes appeared in strip-like forms along minor entrances or road edges of the village.

The “three-core multi-point nesting” pattern reflects the village’s organic adaptation to terrain and hydrology, as well as its socio-cultural logic of spatial usage. The three high-density cores correspond to historical functional centers within the three natural sub-villages—such as defensive watch (e.g., gate towers, night-watch houses), recreational activities (e.g., opera stages), and ancestor worship (e.g., ancestral halls). This spatial structure emerged as people of the same clan settled together around family-based gatehouses and ancestral halls, eventually forming several densely populated neighborhood blocks. These areas exhibit distinct cultural traits such as kinship cohesion, residential clustering, spatial enclosure, and structural stability^77^. Secondary clusters around village entrances and temples are organized around features like bridges, pavilions, pagodas, and temples. This layout likely follows the traditional *feng shui* principle of “water brings wealth,” commonly observed in southern Hunan. Locals believe that rivers symbolize prosperity; thus, architectural elements such as bridges, towers, and pavilions were constructed at river entrances to both shield against wind and rain and to block negative energy, while also retaining wealth within the village^79^. In contrast, the peripheral areas of the village serve defensive and ecological buffering functions, where landscape elements such as ancient walls, stele forests, and commemorative old trees are distributed. Due to their isolated placement and wider spatial dispersion, these areas generally exhibit lower densities of cultural landscape genes.

#### Spatial accessibility analysis of cultural landscape genes

**Fig. 10:**
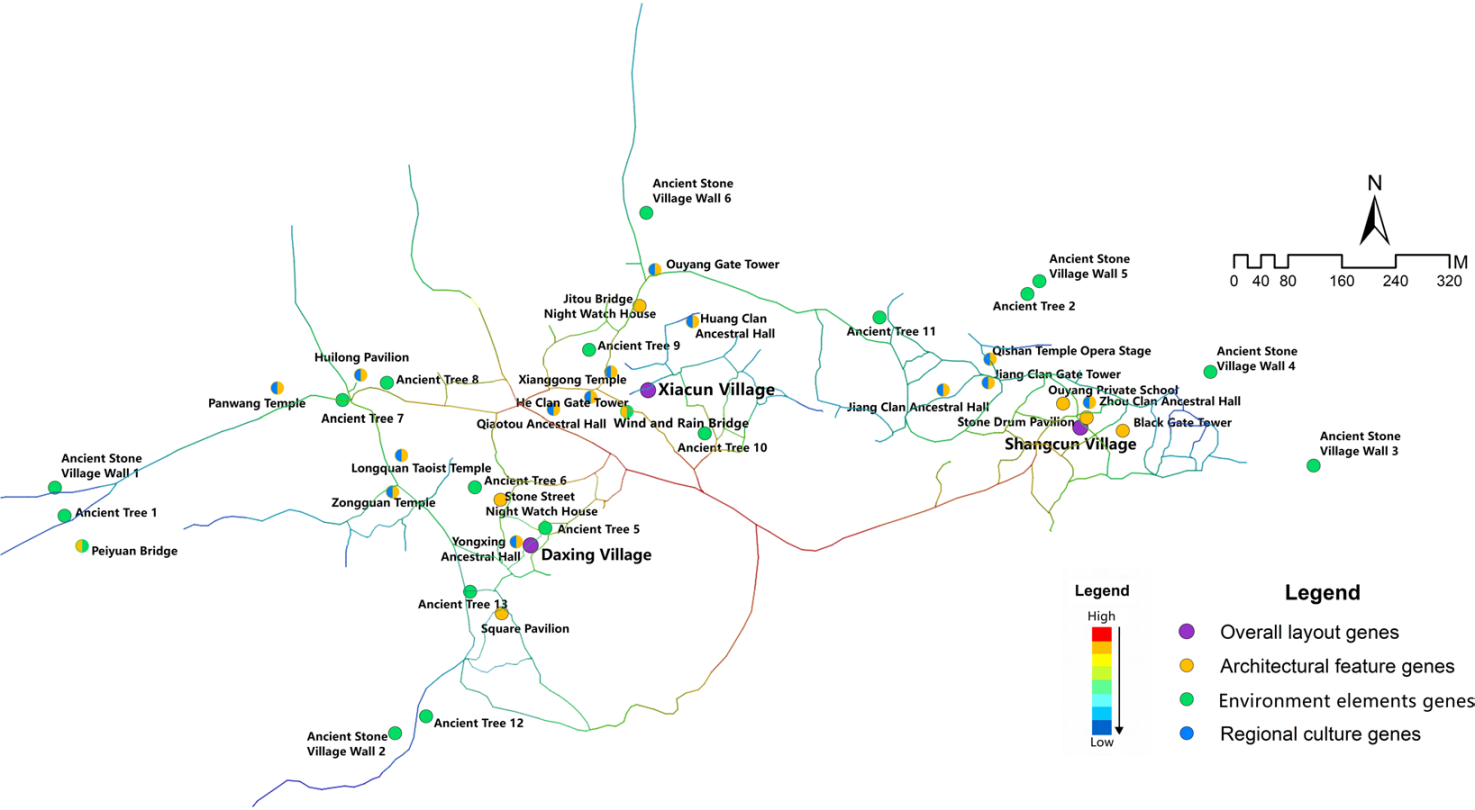
Spatial Accessibility Analysis Results of Goulan Yao Village. Map generated by the author using DepthmapX 0.8.0 (UCL Bartlett Space Syntax, https://github.com/SpaceGroupUCL/depthmapX).

The results of spatial accessibility analysis reveal a distinct “center-to-periphery attenuation structure” in Goulan Yao Village. In Fig. 10, spatial accessibility values range from 0.00 to 1.00 and are represented using a continuous gradient color scale rather than discrete classifications. The color spectrum transitions smoothly from dark blue (very low accessibility) through light blue, cyan, green, yellow, orange, and up to red (very high accessibility). The gradient visually conveys the center-to-periphery attenuation of accessibility across the village, facilitating intuitive interpretation of spatial connectivity variations. High-accessibility zones, represented in red to orange, are mainly concentrated in the central and east-central areas, aligned with the main roads and village core zones—these are the most efficient areas for visitors to access upon entry. Moderate-accessibility zones, shown in yellow to green, are primarily distributed along the village periphery and secondary roads, while low-accessibility zones, marked in blue, are located at the edges of the village or in relatively isolated nodes, indicating poor connectivity and limited accessibility for visitors.

However, the analysis also reveals some structural mismatches: regions with the highest accessibility (red areas) contain relatively few cultural landscape genes. In particular, these zones lack diverse cultural landscape types, with only a few environmental genes (green) and occasional architectural genes (yellow) present, failing to adequately meet visitors’ expectations for immersive cultural engagement. In contrast, some zones with dense concentrations of cultural landscape genes (such as the areas surrounding Daxing Village and Shangcun Village) fall within moderate to low accessibility regions. Specifically, temples, ancestral halls, and clan gatehouses are often located in blue or green zones, leading to a mismatch between cultural richness and spatial reachability.

**Fig. 11:**
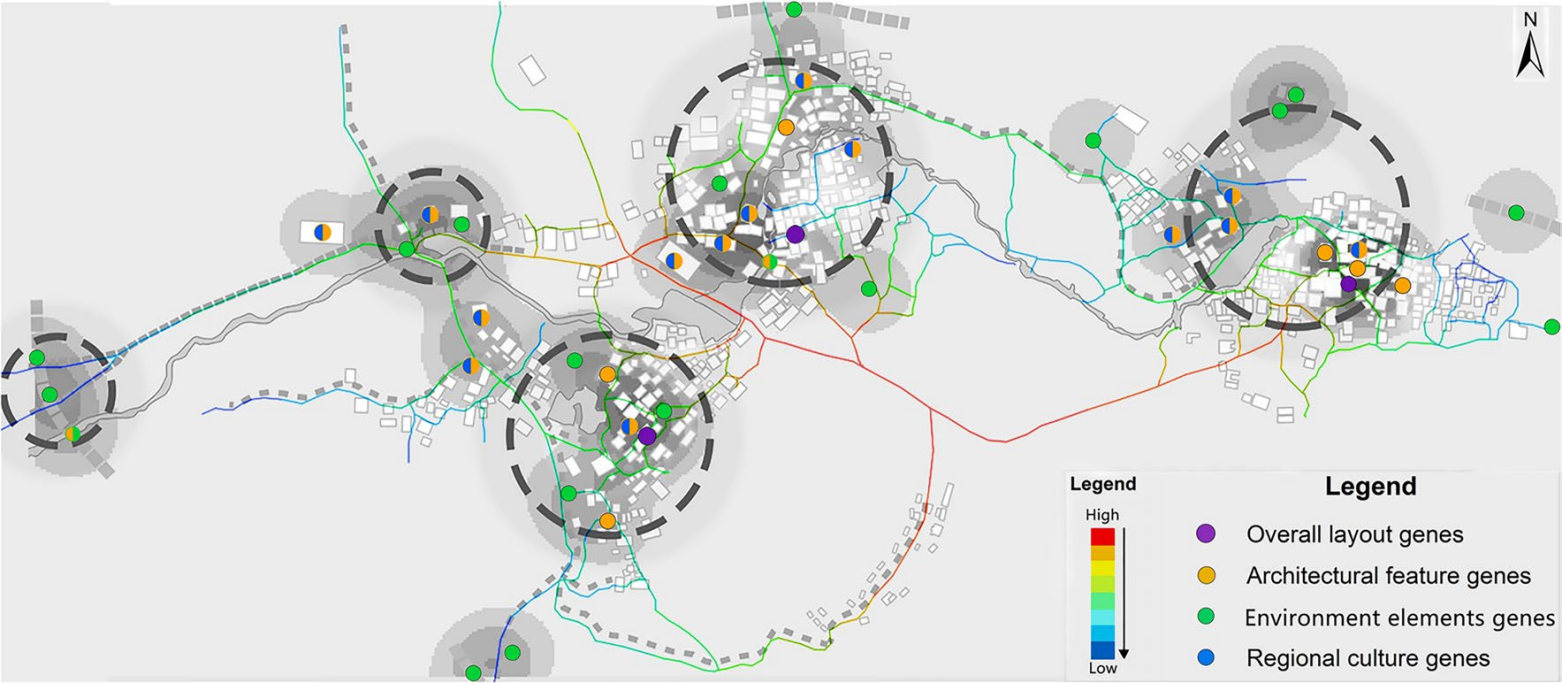
Overlay map of spatial accessibility and kernel density of cultural landscape genes. Map generated by the author using DepthmapX 0.8.0 (UCL Bartlett Space Syntax, https://github.com/SpaceGroupUCL/depthmapX).

As illustrated in Fig. 11, which overlays spatial accessibility analysis with the kernel density distribution of cultural landscape genes, a clear spatial mismatch emerges—characterized by “high accessibility but low cultural density” and vice versa. The observed configuration is primarily rooted in the village’s inherent spatial layout and defensive settlement patterns. On one hand, areas with high concentrations of cultural landscape genes are typically located within the core residential zones, where traditional dwellings cluster densely and the internal circulation system is intricate. The main roads entering the village form a Y-shaped bifurcation, with two north–south axes linking the three natural sub-village units. Within these clusters, numerous secondary roads form consecutive Y-shaped branches, creating a network of narrow, winding, and intersecting paths. This spatial configuration enhances defensive performance by generating confusing turn points that disorient outsiders and make the village more resistant to invasion. However, this same complexity also restricts vehicular access and the installation of modern infrastructure, thereby reducing the accessibility of culturally significant nodes. On the other hand, highly accessible areas are often situated near village entrances or along primary roads, where the terrain is more open and transportation is convenient. These zones are naturally prioritized for logistical functions such as visitor reception, transportation hubs, and service facilities. Since these areas historically served as transitional gateways rather than cultural cores, they contain fewer significant heritage elements, resulting in efficient spatial usage but limited opportunities for immersive cultural engagement.

As a consequence of this spatial-experiential imbalance, many culturally rich areas are overlooked by visitors, leading to diminished satisfaction and shallower cultural engagement. This judgment is further supported by preliminary field investigations involving 5 tourists and 1 local guide, which revealed that visitors tend to concentrate in easily accessible locations such as the wind-rain bridge and Qiaotou ancestral hall, while easily overlooking some culturally dense but locations with poor transportation access (e.g., the Qishan Temple opera stage and the Ouyang private school). Several respondents expressed disappointment that “Some of the interesting places weren’t on the main path,” or that “We saw many houses, but didn’t know which ones were important” These perceptual accounts underscore the experiential impact of spatial mismatch. Meanwhile, the limited visibility and access to heritage sites also weaken community investment in their upkeep, as noted by local village committee members and elderly residents. Consequently, the long-term sustainability and utilization potential of heritage resources is significantly compromised by this spatial-experiential misalignment.

## Discussion

Based on the theoretical framework of cultural landscape genes and the methods of gene identification and coding, this study constructs an index system for landscape gene recognition and a gene information chain map for Goulan Yao Village. Coupled with spatial analysis techniques—including spatial distribution type, density, and accessibility—this research provides an in-depth analysis of the landscape content and spatial characteristics of this traditional Yao village. The findings indicate that:

1. The cultural landscape gene system of Goulan Yao Village demonstrates a rich structural composition and spatial organization. Through the construction of the landscape gene information chain, the study reveals the cultural core composed of natural environment, religious beliefs, and customary practices. The diverse religious belief system and festive activities such as the Panwang Festival and Mud Washing Festival constitute unique carriers of intangible culture. However, the local traditional culture is facing a crisis of transmission, manifested in the weakening of clan consciousness, the difficulties of intangible cultural heritage inheritance, and the insufficient display of cultural elements—modern challenges that urgently require attention.
2. The landscape information points in Goulan Yao Village form a composite cultural spatial system centered on residential buildings, public architecture, and defensive structures. Spatial analysis shows a significantly clustered distribution of cultural landscape genes (ANN = 0.686), forming a “three-core, multi-point nested” spatial pattern. High-density areas are mainly located in the centers of the three natural villages and along the river, while secondary clusters are found in the western part of the village. However, there exists a spatial mismatch between zones of high accessibility and areas rich in cultural resources, creating a structural contradiction between visitor experience and resource conservation. This is detrimental to the sustainable utilization of heritage values.
3. The unique Y-shaped corridor system forms the spatial framework of the landscape in Goulan Yao Village, with the main corridor linking key cultural nodes and the secondary corridors connecting architectural clusters, creating an organically growing spatial structure. This tree-like network demonstrates the spatial organizational wisdom of Yao settlements and contributes to a hierarchical and multi-dimensional cultural landscape system. Nevertheless, some corridors have suffered from overdevelopment or lack of maintenance, restricting the transmission of cultural value. Systematic planning and regulation are needed to enhance the completeness of cultural expression.

### Sustainable revitalization strategies for Goulan Yao Village based on cultural landscape genes

At present, the practical application of landscape gene theory remains in an early exploratory stage^79^. Existing studies primarily emphasize the identification and preservation of landscape genes, while strategies for revitalization and active use remain underdeveloped^80^. In the context of Goulan Yao Village, prior spatial analyses reveal a pronounced mismatch between culturally dense areas and zones of visitor accessibility, resulting in fragmented spatial experience and weakened cultural perception. This spatial-cultural disjunction has emerged as the most pressing challenge to sustainable heritage use and meaningful visitor engagement.

To address this core issue, based on previous comprehensive analysis, this study proposes a revitalization strategy centered on resolving spatial fragmentation and improving cultural legibility, aiming to reintegrate scattered cultural landscape resources and enhance their accessibility and interpretability. The strategy unfolds along four progressive pathways, all designed around this central concern:

1. Preserving cultural connotation, by reinforcing the symbolic integrity of key landscape elements and restoring deteriorated heritage nodes in marginal areas;
2. Optimizing spatial patterns, through improving way finding, enhancing spatial connectivity between key cultural clusters, and redesigning circulation to guide visitors into deeper cultural zones;
3. Developing cultural tourism features, by embedding interpretive systems and cultural narratives into overlooked heritage spaces to enrich visitor experience;
4. Enhancing industrial economy, through small-scale creative industries rooted in landscape genes, thereby reinforcing the cultural economy without compromising spatial authenticity.

Together, these strategies aim to overcome the core challenge of spatial-cultural disjunction, ultimately constructing a revitalization model that integrates heritage conservation with dynamic rural development.

#### Preserving cultural connotation: building a multi-level cultural landscape gene protection system

Preserving the cultural landscape connotation of Goulan Yao Village should begin with establishing a complete archival database of cultural landscape genes^32^, systematically recording the spatial positions, historical values, and cultural meanings of various landscape information elements. On this basis, a graded protection strategy is proposed: core landscape gene elements such as Panwang Temple and Shigu Pavilion, which hold significant historical and cultural value, should be strictly protected to maintain their authenticity, and damaged ancestral halls and defensive structures should be repaired. Auxiliary elements such as traditional residential buildings and daily-life spaces can be moderately activated and utilized, introducing modern functions while retaining traditional forms to organically combine cultural inheritance with functional renewal. For intangible cultural heritage, a complete “record–transmit–display–innovate” protection chain should be built, supporting the inheritors of traditional crafts and encouraging integration between Long Drum Dance, Yao Embroidery, and contemporary design. In parallel, digital technologies can be employed to create a “cultural memory bank” for Goulan Yao Village, using panoramic photography, 3D reconstruction, and other methods to virtually preserve and disseminate endangered cultural landscapes. More importantly, the construction of a community participation mechanism should be strengthened, turning local residents into primary actors in cultural protection. Through grassroots self-organization, endogenous motivation can be stimulated, shifting the paradigm from external intervention to internal development^81^.

#### Optimizing spatial patterns: reshaping the continuity and accessibility of landscape genes

**Fig. 12:**
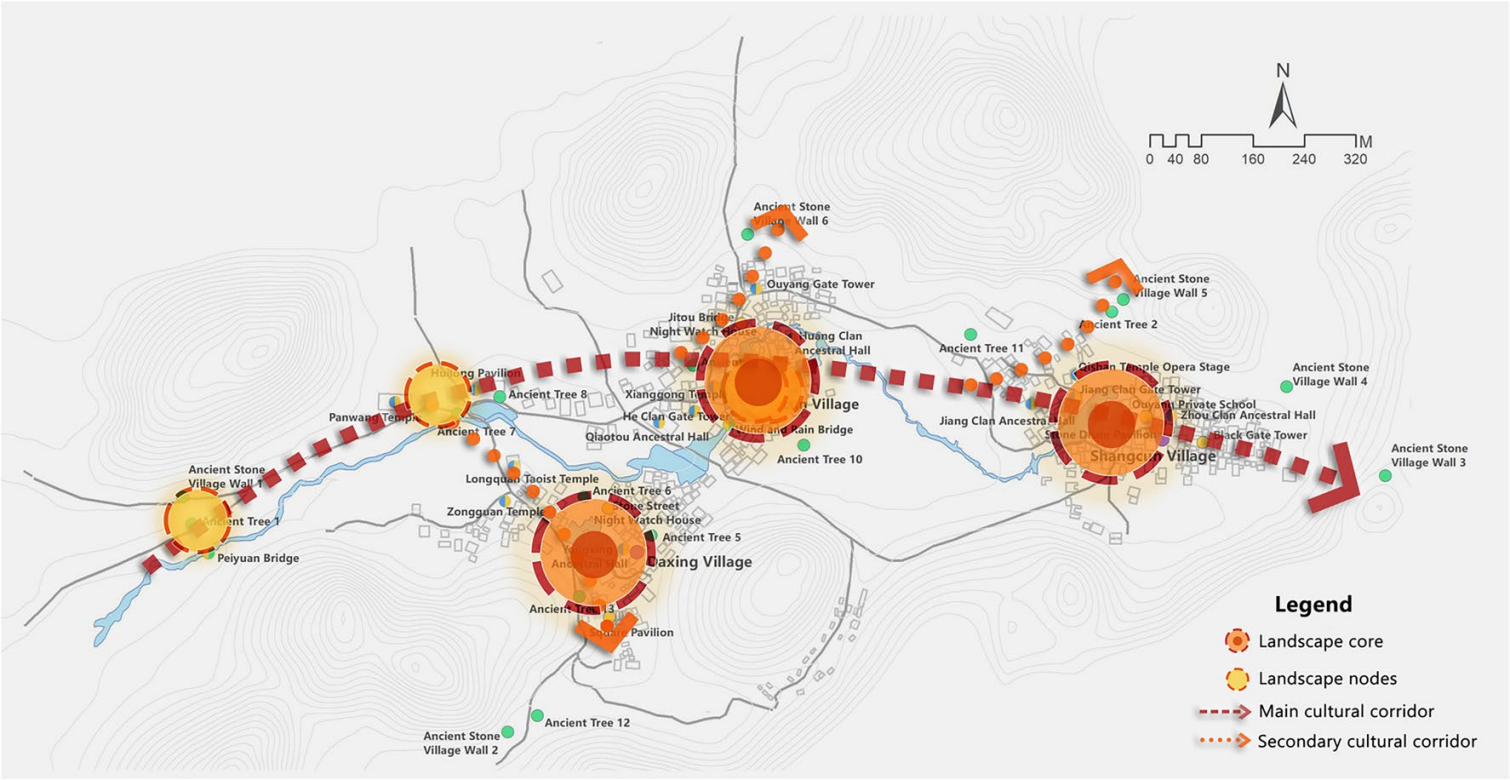
Landscape spatial conservation model for Goulan Yao Village. Map generated by the author using ArcGIS 10.8 (Esri Inc., https://www.esri.com/en-us/arcgis/products/arcgis-desktop/overview).

Comprehensive planning of the spatial distribution of landscape resources in Goulan Yao Village is essential for their effective protection and utilization^82^. Based on the results of kernel density and accessibility analyses, the spatial distribution characteristics of landscape nodes are clearly identified, providing scientific evidence for resource protection. The study finds that the Y-shaped main corridor constitutes the cultural axis of Goulan Yao Village, connecting most of the landscape gene elements. Accordingly, the innovative concept of a “cultural landscape gene corridor” is proposed. By integrating pedestrian routes, rest nodes, and cultural display platforms, four tourism-oriented corridors are planned to construct an organic network connecting all cultural landscapes and forming a “point–line–plane” multi-dimensional experience system (Fig. 12). This strategy not only improves the cultural landscape experience of visitors but also promotes spatial integration and balanced development, achieving harmony between protection and utilization.

For the three core nodes with high densities of landscape elements, development priorities should be assigned, with a focus on enhancing public infrastructure and implementing appropriate landscape improvement designs. In response to the current imbalance in the spatial distribution of cultural landscape genes and the mismatch in accessibility, we propose reconstructing the spatial network of the landscape and optimizing the “accessibility–density” spatial structure. The foremost task is to restore the broken cultural landscape corridors, particularly the primary corridors that serve as structural backbones. It is also essential to modify modern constructions that disrupt spatial continuity, in order to maximize the recovery of traditional alleyway patterns. In areas rich in cultural resources but with low accessibility, connectivity can be improved through micro-scale environmental interventions and path system optimization. At the same time, the secondary passage network should be enhanced, with additional directional signage systems installed to improve the identifiability and accessibility of peripheral landscape nodes.

#### Developing cultural tourism characteristics: activating the experiential value of cultural landscape genes

Based on the analysis of cultural landscape genes in Goulan Yao Village, we propose establishing a tiered experiential system anchored in three implementable strategies to activate tourism potential while ensuring cultural authenticity. First, by enhancing the annual festival activities, the experiential value of cultural landscape genes can be activated and broader visibility achieved. This includes organizing curated cultural performances at multiple locations across the village, such as Panwang Festival worship ceremonies, Long Drum and folk song performances, as well as the locally distinctive traditional agrarian celebration—the Mud Washing Festival. Second, a heritage education program titled “Yao Living Classroom” can be developed for school groups and cultural tourists. The program would offer structured half-day to multi-day modules, including guided visits to cultural heritage sites within the village, followed by hands-on workshops led by local artisans on Yao Embroidery, Yao herbal medicine, or Yao women’s boxing. Visitors could also experience Goulan Yao Village’s traditional cuisine, attire, and festival practices. Overnight stays can be arranged in homestays converted from historic dwellings, where participants can taste traditional foods such as pounded glutinous rice cakes, stuffed bitter melon, steamed bean rice, and Yao-style oil tea. These activities would deepen immersive engagement and cultural awareness, safeguarding spatial heritage while moving beyond the superficial “sightseeing-only” tourism model—thus enriching the cultural dimension of the local tourism economy. Third, to promote environmentally sensitive and culturally immersive experiences, a “Yao Slow-Travel System” can be implemented, consisting of clearly marked walking trails along the village’s three natural settlement clusters. The system will integrate hand-drawn route maps, landscape interpretation signage, and rental services for audio guides and local bicycles. QR codes at trail nodes can provide AR-enhanced content—such as historical reconstructions of village gates or narrated Yao myths—allowing low-impact, high-engagement exploration. These three interconnected projects aim to create a sustainable tourism framework that supports cultural revitalization, fosters local participation, and enhances visitor immersion in the unique Yao landscape system.

#### Enhancing industrial economy: constructing a value transformation mechanism for cultural landscape genes

Grounded in the systematic protection and revitalization of cultural landscape genes, a diversified industrial development model should be established to achieve the sustainable economic development of traditional villages. Leveraging Goulan Yao Village’s ethnic cultural landscape resources, a “culture + tourism + creativity” industrial chain can be built to cultivate boutique homestay clusters with Yao characteristics, develop signature cultural and creative products, and form distinctive industry clusters. In terms of industrial organization, the establishment of villagers’ cooperatives or community enterprises is encouraged. By adopting equity-sharing models, villagers can directly participate in tourism development and profit distribution, thereby creating an effective benefit-sharing mechanism^81^. Additionally, a “cultural landscape gene + internet” digital economy model can be constructed to build a digital cultural platform for Goulan Yao Village. Through online exhibitions, live-streamed marketing, and crowdfunding for conservation, the platform would expand both cultural influence and economic return. Special attention should be paid to fostering synergies across sectors—for instance, integrating traditional Yao agriculture with rural tourism to develop agritourism and branded specialty products; or transforming traditional craft skills into creative design and educational experiences, thereby achieving both heritage transmission and economic gains. Through multidimensional industrial integration, a unique industry system centered on cultural landscape genes can be constructed, driving traditional villages toward a truly sustainable development path.

This strategy aligns with international practices in cultural heritage villages. For instance, Miyama Village in Japan has successfully preserved its thatched-roof houses (kayabuki) through a model combining eco-tourism, community-run homestays, and craft-based education programs^83^. Similarly, Hahoe Village in South Korea integrates Confucian cultural tourism with traditional mask dance performances and family-run tourism businesses. In addition, to sustainably manage the historic village, the government actively supports the living conditions of local residents—preserving their lifestyle, customs, traditions, and overall quality of life^84^. This approach has achieved a win-win outcome of cultural preservation and income generation. These international cases demonstrate that deeply integrating cultural heritage resources with creative industries—while ensuring equitable community participation and benefit-sharing—is essential for the sustainable revitalization of traditional villages. Drawing inspiration from Miyama and Hahoe, Goulan Yao Village can establish a diversified industrial system centered on cultural landscape genes. This system would foster synergies among Yao traditional agriculture, folk festivals, and rural handicrafts. Moreover, by encouraging the formation of villagers’ cooperatives, local residents can directly participate in operating homestays, craft workshops, and cultural events—enhancing both cultural agency and economic benefits. Such a model enables cultural revitalization and industrial co-development, forming a foundation for long-term sustainability.

### Theoretical contributions

Within the framework of cultural landscape gene theory, this study systematically explores the spatial structure and cultural connotation of traditional Yao villages by integrating gene identification with spatial analysis methods. It enriches the practical application path of landscape gene theory in minority ethnic village contexts and makes several theoretical contributions to the field of cultural landscape gene theory and traditional village conservation.

1. This research innovatively integrates the landscape gene information chain with spatial analysis techniques, constructing a “gene identification–spatial interpretation–revitalization strategy” framework, thereby expanding the methodological dimensions of cultural landscape research in traditional villages.
2. In contrast to existing literature that tends to emphasize material genes^85^, this study reveals a triadic cultural core in Goulan Yao Village comprising the natural environment, belief systems, and customary practices. This deepens the understanding of interaction mechanisms between intangible landscape genes and material carriers, adding a new layer to the conceptual system of cultural landscape genes.
3. In response to the prevailing tendency in rural cultural conservation to emphasize static preservation while neglecting dynamic revitalization^86^, this study proposes a “four-dimensional pathway” strategy that integrates gene conservation with spatial planning, cultural tourism experiences, and industrial economy. This approach advances the transformation of landscape gene theory from cultural interpretation to practical application. These theoretical developments not only enrich the applied dimensions of cultural landscape gene theory but also offer a replicable analytical framework for the protection and revitalization of similar traditional villages.

### Limitations and future research

Despite progress in identifying cultural landscape genes, conducting spatial analysis, and formulating revitalization strategies for Goulan Yao Village, this study has limitations. Focusing on a single case and relying on static spatial data limits the generalizability and temporal depth of the findings. To enhance theoretical transferability, future research could adopt a comparative design across Yao or Tujia villages with varied geomorphological settings, settlement forms, or preservation levels, testing the adaptability of the “gene–space–strategy” framework. Due to time and access constraints, certain stakeholder groups—particularly intangible cultural heritage bearers and younger villagers—were underrepresented, potentially limiting cultural insight diversity; future studies should prioritize broader engagement. The mechanisms behind gene evolution also require deeper investigation, especially the role of tourism in transforming or commercializing landscape genes. This could be addressed through historical GIS, remote sensing, and time-series analysis to trace changes before and after major tourism interventions. Integrating digital tools such as AR-enhanced tours or digital twin systems may further improve real-time interpretation, community interaction, and cultural activation. These directions will strengthen the framework’s cross-case applicability, temporal richness, and technical innovation.

## Conclusion

This study advances the theory and application of cultural landscape genes by systematically integrating gene identification with spatial analysis in the context of Goulan Yao Village. Theoretically, it proposes a “gene–space–strategy” analytical model that deepens the understanding of how intangible cultural expressions interact with spatial form, moving beyond a material-centric perspective. This study further expands the research paradigm of landscape genes by focusing on mountainous Yao villages—a settlement type that has been insufficiently explored, characterized by fortress-like defensive landscapes and clan-based spatial clustering. Methodologically, the study innovatively integrates cultural landscape gene theory with GIS-based spatial analysis—including kernel density, accessibility, and clustering assessments—to interpret the spatial logic of traditional villages. This interdisciplinary approach provides a replicable analytical pathway for research on ethnic cultural landscapes. Practically, it proposes a four-dimensional revitalization strategy—preservation, spatial optimization, cultural tourism, and industrial integration—anchored in the unique cultural system of the Yao people. These findings respond directly to China’s ongoing rural revitalization strategy and the national agenda for the protection and development of ethnic minority cultural heritage. By aligning landscape conservation with local livelihoods and community-led tourism, the study offers policy-relevant insights for integrated rural governance, while encouraging active participation of local residents in both planning and implementation. The results further yield theoretical depth, methodological adaptability, and actionable guidance for the sustainable renewal of traditional ethnic villages.

It should be noted that this study is primarily based on static spatial analysis and does not adequately address the diachronic evolution of landscape genes. It focuses on a single case and relies on static spatial data, which may affect the broader applicability and temporal depth of the findings. Future research could include comparative studies with other Yao or Tujia villages to test the framework’s generalizability. Additionally, incorporating dynamic methods such as historical GIS, UAV mapping, or AR-based interpretation could help capture changes over time and enhance visitor engagement. These directions would strengthen both the analytical depth and practical relevance of cultural landscape research in traditional villages.

## Supplementary Information

Supplementary Information 1.

Supplementary Information 2.

Supplementary Information 3.

Supplementary Information 4.

Supplementary Information 5.

Supplementary Information 6.

## Data Availability

Data are contained within the article.
