# Supplementary figures and images for: Formulating sustainable planning for Goulan Yao Village based on the integration of cultural landscape gene theory and spatial analysis
Source: Sci Rep. 2025 Aug 14;15:29872. doi: 10.1038/s41598-025-15357-2 (PMC12354706; doi:10.1038/s41598-025-15357-2)

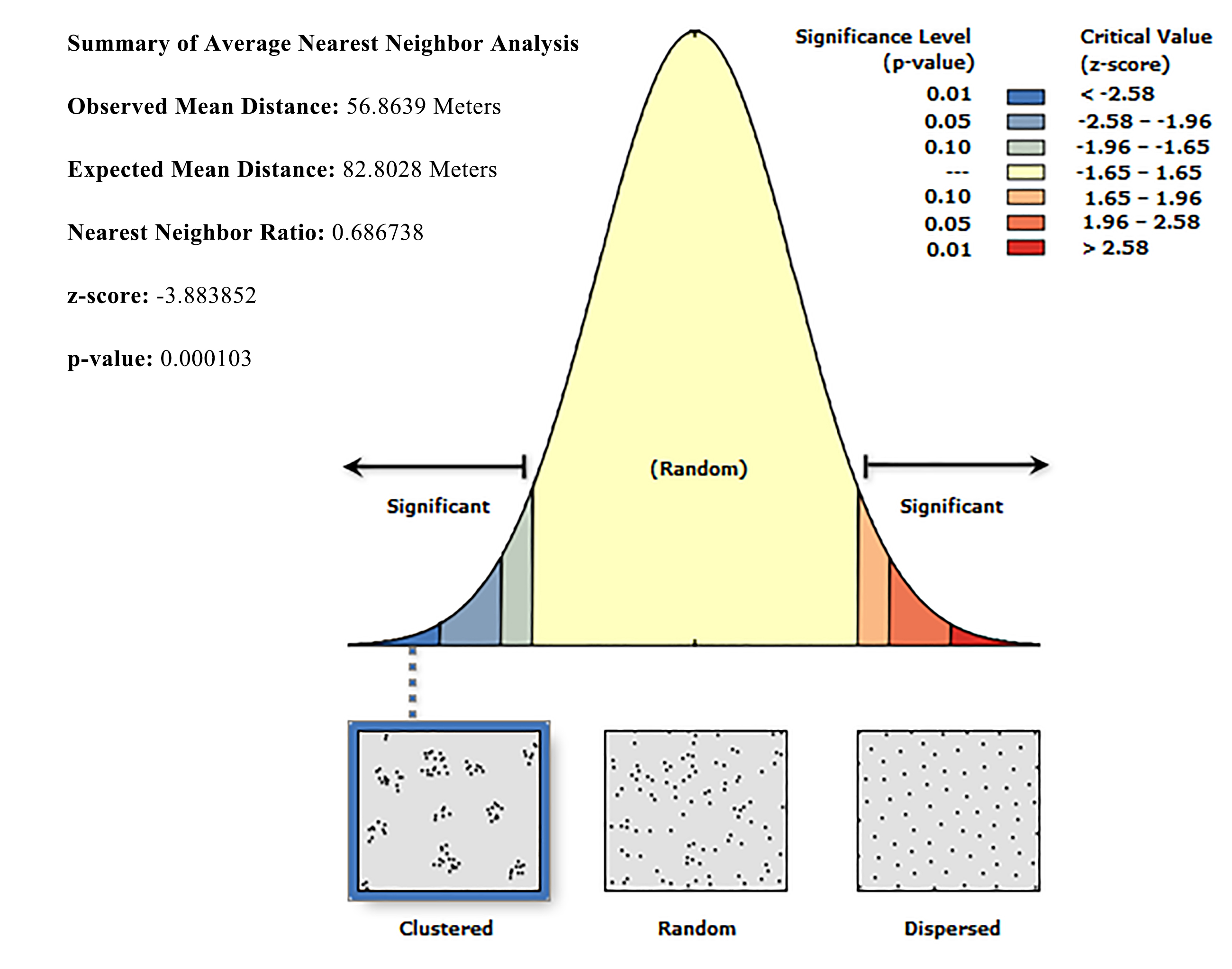

Supplement: Supplementary file 1 — Supplementary Information 1. [file 41598_2025_15357_MOESM1_ESM.jpg]

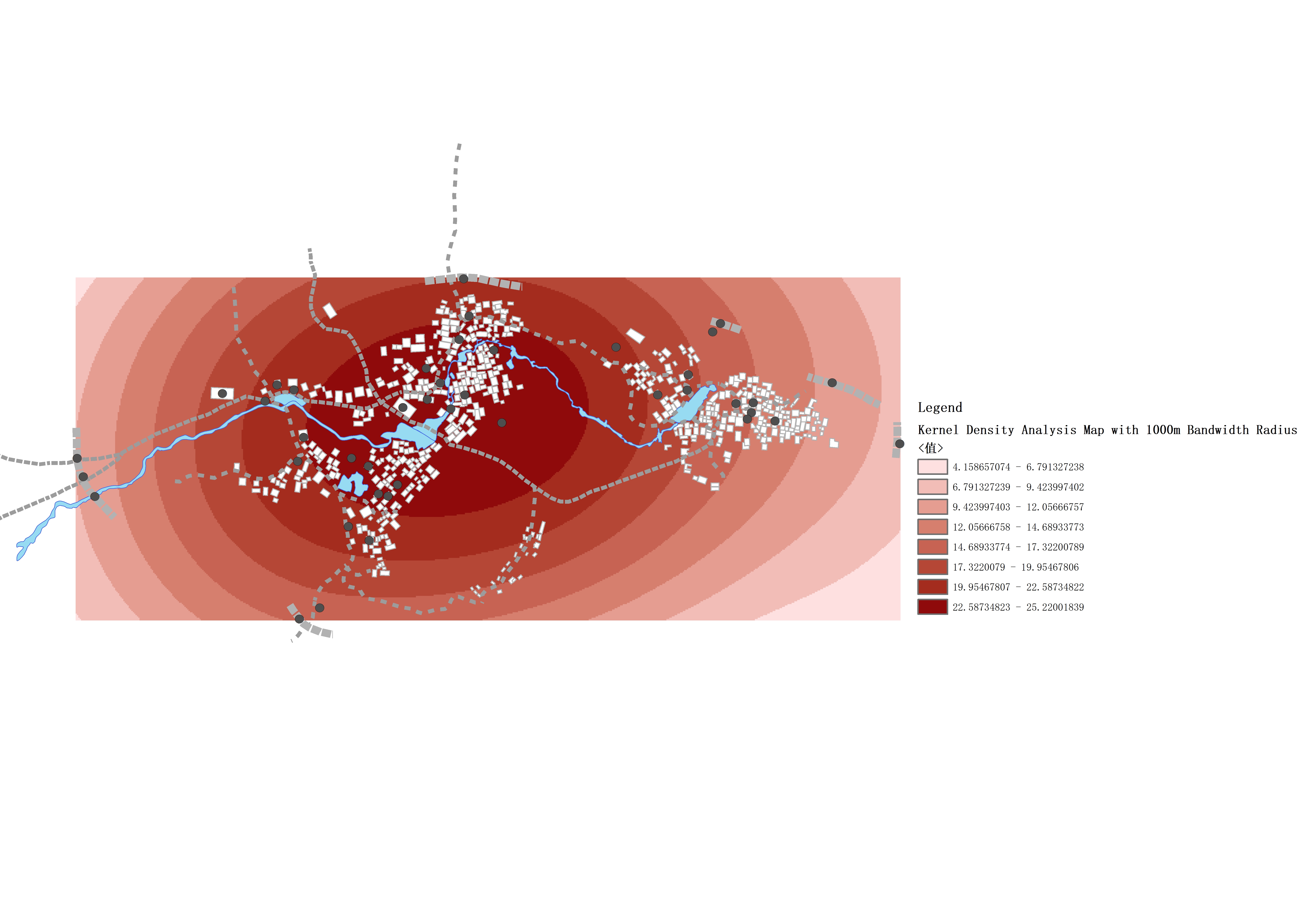

Supplement: Supplementary file 3 — Supplementary Information 3. [file 41598_2025_15357_MOESM3_ESM.jpg]

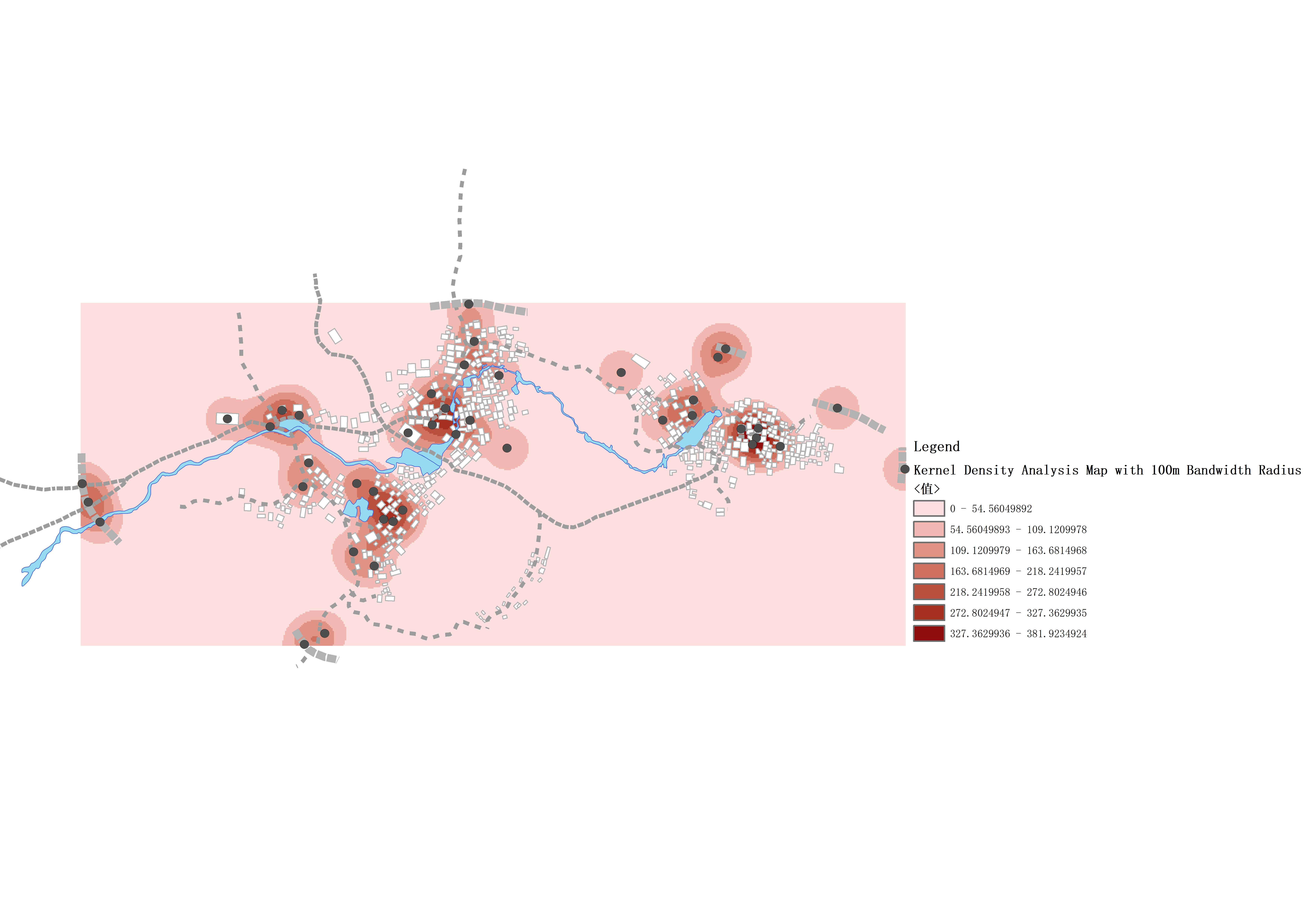

Supplement: Supplementary file 4 — Supplementary Information 4. [file 41598_2025_15357_MOESM4_ESM.jpg]

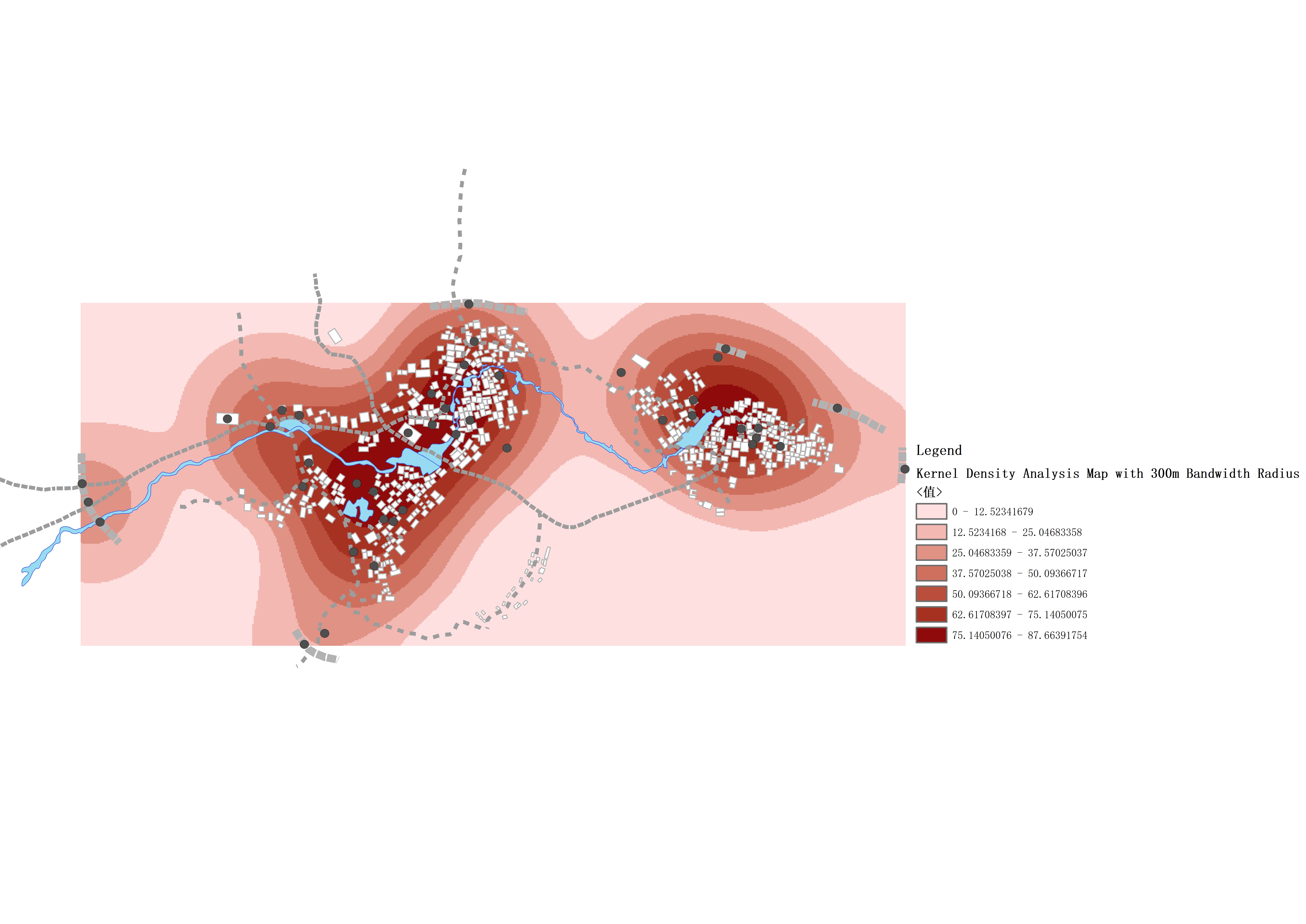

Supplement: Supplementary file 5 — Supplementary Information 5. [file 41598_2025_15357_MOESM5_ESM.jpg]

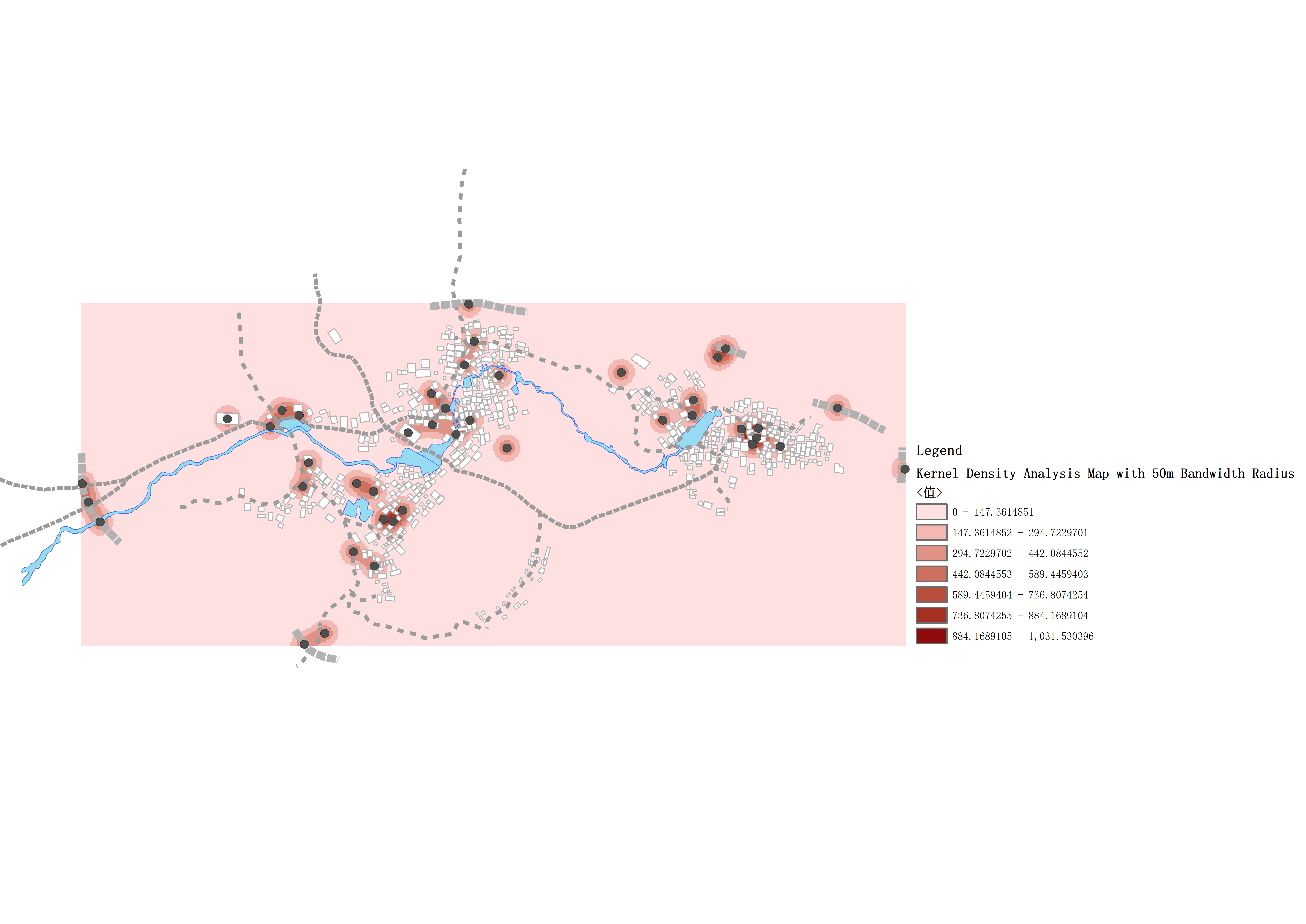

Supplement: Supplementary file 6 — Supplementary Information 6. [file 41598_2025_15357_MOESM6_ESM.jpg]
